# Computational Studies of the Dynamics of the Human Iron Exporter Ferroportin and Insight into the Role of Crucial Residues Involved in Pathogenic Mutations

**DOI:** 10.3390/biom16101382

**Published:** 2026-09-22

**Authors:** Sergio Vulterini, Maria Carmela Bonaccorsi di Patti, Giovanni Musci, Fabio Polticelli

**Affiliations:** 1 Department of Sciences, Roma Tre University, 00146 Rome, Italy; sergio.vulterini@uniroma3.it; 2 Department of Biochemical Sciences ‘A. Rossi Fanelli’, Sapienza University of Rome, 00185 Rome, Italy; mariacarmela.bonaccorsi@uniroma1.it; 3 Department of Biosciences and Territory, University of Molise, 86090 Pesche, Italy; giovanni.musci@unimol.it

**Keywords:** hemochromatosis, ferroportin, iron, molecular dynamics

## Abstract

Ferroportin (FPN/SLC40A1), the only known human iron exporter, plays a key role in iron homeostasis through an alternating access mechanism involving inward–open, occluded, and outward–open conformations. Pathogenic FPN mutations cause ferroportin–related iron overload, an autosomal dominant iron overload disease characterized by early–onset anemia, hepatic fibrosis, and diabetes. This disease can be further categorized into two subtypes: ferroportin disease, characterized by low transferrin saturation (TSAT) and iron overload predominantly in Kupffer cells, and ferroportin–related hemochromatosis, defined by high TSAT and iron overload in hepatocytes. However, the structural and functional characteristics governing FPN’s conformational transitions and their relationship to pathogenic mutations remain poorly understood. Therefore, we conducted comprehensive modeling and molecular dynamics studies to investigate the conformational states of the transporter and analyze the molecular basis of disease–causing mutations. Our analysis revealed shared dynamic properties across FPN conformations, with all the three main conformations displaying stable protein cores surrounded by dynamic peripheral regions. We observed significant differences in the most stable interactions established between the two lobes of the transporter across the three conformational states, enabling identification of key residues involved in state–dependent stabilization. These findings were interpreted in the context of known pathogenic mutations to assess their potential effects on protein stability and transport mechanism. This approach provides crucial insights into the molecular determinants of FPN function and dysfunction, offering a foundation for understanding how specific mutations disrupt its specific conformational states and lead to ferroportin–related iron overload. The identification of conformationally important residues enhances our understanding of FPN’s transport mechanism and provides a framework for interpreting the pathological consequences of genetic variants in iron metabolism disorders.

## 1. Introduction

Iron is an essential transition metal involved in many crucial biological functions, such as oxygen and electron transport, DNA repair, and the homeostasis of the nervous and cardiocirculatory systems [1]. Alongside these physiological functions, there are pathological effects deriving from the dysregulation of iron homeostasis, where the presence of the free-redox ion leads to the production of ROS (reactive oxygen species) via Fenton reactions and the resulting indiscriminate oxidation of biomolecules. For this reason, intracellular and extracellular levels of iron must be tightly regulated, and this is achieved by the presence of a transport system that involves a great variety of iron importers and a few iron exporters [2]. To this day, the only known iron exporter in cells is ferroportin (FPN), a protein belonging to the SLC40 family, which in turn is part of the Major Facilitator Superfamily (MFS), the largest known protein family of secondary active transporters [3]. Also known as SLC40A1, FPN shares the fold of most of the MFS members, with 12 transmembrane α–helices (TM) organized in an inverted and repeated “3-plus-3” form making up an N–lobe and a C–lobe. TM1, TM2, and TM3 are symmetrically related to TM4, TM5, and TM6 in the N–lobe by a 180° rotation around an axis parallel to the plasma membrane. Similarly, TM7, TM8, TM9 are related to TM10, TM11, and TM12 in the C–lobe. Furthermore, the iron transporter structure comprises two long loops that connect TM6 and TM7 on the intracellular side (intracellular loop 3 or ICL3) and TM9 and TM10 on the extracellular side (extracellular loop 5 or ECL5). Despite all these similarities, FPN displays some unique features inside the superfamily, namely, the separation of the TM7 α–helix into two smaller α–helices, TM7a and TM7b, and the existence of two substrate-binding sites instead of one. These are formed by the residues couples D39–H43 in the N–lobe (“S1”) and C326–H507 in the C–lobe (“S2”) [4].

FPN-mediated iron transport occurs via an alternating access mechanism based on the rigid body rotation of the N–lobe and the C–lobe of the protein [5], with the protein cycling from an initial inward–open conformation, open towards the intracellular side, through an occluded and a final outward–open conformation, which allows the release of the transition metal on the extracellular space. The transport mechanism in human FPN (hFPN) requires a rearrangement of the ionic interactions and hydrogen bonds between a first set of helices formed by TM1, TM4, TM7, TM10 and a second set formed by TM2, TM5, TM8, and TM11. This reorganization allows the formation of an intracellular gate that stabilizes the outward–open conformation thereby finalizing the iron transport cycle [6,7]. In a bacterial homolog of hFPN, namely, the iron exporter BbFPN from *Bdellovibrio bacteriovorus*, the stabilization of the inward–open conformation has been attributed to the formation of a hydrophobic interactions-based extracellular gate involving helices TM1, TM7, and TM11 [8]. The Fe^2+^ transport mechanism is chemically electroneutral because hFPN couples the iron export to the import of two H^+^ ions in the cytoplasm [4], although contrasting evidence has been published in support of a Fe^2+^/2H^+^ symport mechanism [9]. Finally, hFPN acts as a Ca^2+^ uniporter too, where the ion acts as an essential cofactor for the iron efflux by favoring the transition from the outward–open to the inward–open conformation, thereby allowing the binding of the metal on the intracellular side [10,11].

Once hFPN releases iron outside of the cell, the ion is subsequently oxidized from Fe^2+^ to Fe^3+^ by ceruloplasmin, a multi-copper ferroxidase that couples the oxidation of iron to the reduction of oxygen into water, and finally released to transferrin [12,13]. In addition, in Mammalia, there are two homologs of ceruloplasmin that also oxidize the Fe^2+^ exiting from hFPN: the first one is hephaestin, which oxidizes iron for its dietary absorption in the enterocytes of the small intestine, while the second one is the more recently discovered zyklopen, which is localized in the placenta. Curiously, the activity of the latter ferroxidase seems also to be crucial for the correct growth of the hair follicle [14,15].

Inside the cell, hFPN receives Fe^2+^ from the chaperone protein PCBP2, previously annotated as an RNA-binding protein, whose structure comprises three KH domains (KH1, KH2, KH3) and that interacts with the C–terminal end of hFPN via the KH2 domain [16,17].

The expression of hFPN in Vertebrata is mainly regulated by the action of hepcidin, a hepatic hormone produced in response to high levels of circulating iron and during inflammation or infection conditions, which binds to the outward–open conformation of hFPN in the S2 iron-binding site and induces the internalization and subsequent ubiquitin-mediated degradation of the iron exporter [18,19]. This process involves the binding of ubiquitin to lysine residues located in the ICL3 and requires the participation of the E1 enzyme UBA6, the E3 enzyme Rnf217, and the adaptor protein NDFIP1 [20,21]. Moreover, being an iron-related protein, hFPN expression is also regulated via the IRP-IRE system, which ensures that hFPN translation, as for other proteins involved in iron metabolism such as ferritin and DMT1, is inhibited in low intracellular iron conditions [2].

Being considered as the key coordinator of the iron balance between intracellular and systemic iron homeostasis [22], it is not surprising that non-sense mutations in the *SLC40A1* gene lead to embryonic death in vivo [23,24]. On the other hand, mis-sense mutations lead to clinical phenotypes such as hepatic fibrosis, diabetes, and early-onset anemia that fall within an iron overload autosomal dominant disease named ferroportin-related iron overload, previously known as hereditary hemochromatosis type 4 [25]. Based on the mutation type and on the phenotypic manifestations, this disease can be further distinguished into two groups with different phenotypes. The first one is the more widespread ferroportin disease (FD), which was previously referred to as hereditary hemochromatosis type 4A. In this disease form, loss-of-function (LOF) mutations, which affect residues located at the cytoplasm−membrane interface, cause iron overload in the reticuloendothelial macrophages and are mostly associated with a reduction in the iron transport and in the membrane localization of hFPN. This disease form is characterized by low-medium transferrin saturation (TSAT) and high ferritin levels [26]. Conversely, the less frequent SLC40A1/ferroportin-related hemochromatosis (SLC40A1-HC), previously known as hereditary hemochromatosis type 4B, is caused by gain-of-function (GOF) mutations, which affect mostly residues involved in the binding of hepcidin and are associated with iron overload in liver hepatocytes, a high TSAT, and high ferritin levels [26,27]. This last phenotype is more similar to the HFE-HC (HFE-related hemochromatosis) [28], which is the most common form of hereditary hemochromatosis caused by mutations in the *HFE* (hereditary hemochromatosis protein) gene. This alteration results in a reduced production of hepcidin from the liver and in a phenotype characterized by high TSAT and serum ferritin, and low spleen iron [29]. In the last reclassification of HC diseases, along with renaming hereditary hemochromatosis type 4 as SLC40A1-related iron overload and the former type 4B as SLC40A1-related hemochromatosis, it has been proposed to abandon the 4A/4B terminology for these two diseases and to exclude ferroportin disease from the known HCs due to its distinct phenotype with respect to the other forms [25,30].

Given the high biomedical relevance of hFPN, we focused on performing a structural and functional characterization of hFPN using an in silico approach. This was necessary for two main reasons: (i) to this day, there are no experimentally determined structures of the inward–open conformation of hFPN, and (ii) the structures of the occluded and outward–open conformations obtained via Cryo-EM do not include the intracellular and extracellular loops of FPN, which instead seem to be essential for the degradation of the transporter [4] and the final phase of the iron export, respectively [31]. More precisely, the open-source software ColabFold v1.6.3 [32] was used to obtain a structural model of the inward–open, occluded, and outward–open conformations of hFPN. These models of hFPN were subsequently used to perform molecular dynamics (MD) simulations to identify the residues that are responsible for the correct global folding of hFPN and the state-dependent structural differences between these three conformations. Finally, data were interpreted based on the hFPN known pathogenic mutations and their possible effect on the stability of the functionally relevant conformational states of hFPN. 

## 2. Materials and Methods

### 2.1. Sequence Analysis

#### 2.1.1. Search of Orthologs of hFPN

The search for orthologs of hFPN (UniProt ID: Q9NP59) was conducted using the protein Basic Local Alignment Search Tool (“BlastP”) algorithm, and the most relevant sequences within the biological kingdoms Animalia and Fungi were selected. The BLAST 2.17.0 algorithm allows protein or nucleotide sequences to be compared with each other based on the presence of regions of local similarity, comparing them with sequences in a database and calculating the statistical significance of the various matches [33]. The sequences were downloaded from the Uniprot database [34].

#### 2.1.2. Multiple Sequence Alignment

The sequences of hFPN and its orthologs were then used to perform a multiple sequence alignment (MSA). For the purposes of the alignment, the sequences showing the highest degree of identity with the hFPN sequence were chosen for each clade.

Multiple sequence alignments were generated using the Clustal Omega v. 1.2.4 program [35] and visualized using the Jalview program and applying the Zappo color scheme [36].

The chosen sequences for the MSA are listed in Supplementary Table S1 and the final MSA in Jalview is shown in Supplementary Figure S1.

### 2.2. Structural Predictions and Models Validation

The whole sequence of hFPN (Uniprot ID: Q9NP59) was used as an input in the open-source modeling software ColabFold [32], an implementation of Alphafold2 that allows various conformations of the same protein to be obtained in a single simulation. This feature was exploited to produce the structural models of the transporter in the inward–open, occluded, and outward–open conformations. ColabFold allows the depth of the MSA and the uncertainty of the models to be increased by varying the value of the max_msa_cluster parameter, which in our case was set to 512–1024 to obtain the structural model of the inward–open conformation, to 16–32 for the occluded conformation, and finally, to 32–64 for the outward–open state of hFPN. The pLDDT and PAE plots for each conformational model of human ferroportin are show in Supplementary Figure S2.

Each hFPN model was subsequently compared to other experimentally structures of ferroportin and other MFS/SLC transporters available on the PDB using the TM-align algorithm [37]. We compared the position of the transmembrane α–helices of hFPN, thus excluding the two intracellular and extracellular loops and the N–terminal and C–terminal ends. This was done because TM-score was designed to be more sensitive to global fold similarity rather than local structural variations when comparing two protein structures. More in detail, the score weights smaller distance errors, i.e., more structured regions, stronger than larger distance errors, i.e., disordered regions such as the two hFPN loops.

The two G80S and N174I mutants were obtained by manually mutating the specified residue in the outward–open conformational model via Swiss-PDB viewer [38].

### 2.3. Molecular Dynamics Simulations

The structural models of the three conformations of hFPN and of the two G80S and N174I mutant proteins were inserted in a phospholipid membrane bilayer mimicking the plasma membrane (see Supplementary Table S2 and Supplementary Figure S3) using as a reference for its composition the plasma membrane model published by the CHARMM-GUI team (https://charmm-gui.org/?doc=archive&lib=biomembrane; last accessed on 11 September 2026) [39]. The system was solvated adding H_2_O molecules on both sides of the membrane to reach a width of 22.5 Å on each side of the membrane. Furthermore, the system was enlarged by a 1.5× factor since the initial system was too small to correctly simulate the protein inside the membrane, given the length of the intra- and extracellular loops. The protonation states for the ionizable residues were assigned using PROPKA [39]. The charge of the whole system was neutralized by adding 0.15 M of NaCl. The CHARMM36m force field [40] and the AMBER22 software package [41] were used to perform the MD simulations. First, an energy minimization procedure that involved 2500 steps of the steepest descent algorithm followed by 2500 steps of the conjugate gradient one was performed. Positional restraints were applied on the protein residues (10 kcal mol^−1^ Å^−2^) and on the membrane (2.5 kcal mol^−1^ Å^−2^). The resulting minimized systems were then simulated using the canonical NVT ensemble, reaching a final temperature of 310.15 K. Thereafter, isothermal–isobaric simulations using the NPT ensemble were performed to equilibrate the pressure to 1 bar. During the thermalization and equilibration phases, the positional restraints were gradually reduced until the equilibrated systems, without restraints, were properly simulated using the NPT ensemble for a total of three replicas of 1 µs for the wild-type systems, 2 replicas of 1 µs for the N174I mutant, and finally, one replica of 1 µs for the G80S mutant. The Langevin thermostat was used for both NVT and NPT ensembles, while for pressure control, the Monte Carlo barostat was used with a semi-isotropic pressure scaling. Each MD simulation was performed in a periodic boundary system. For the long range non-bonded interactions, the Particle Mesh Ewald method with a 12 Å cut-off was used. The MD simulations were performed with a time step of 2 fs.

### 2.4. Molecular Dynamics Analysis

The simulation results were visualized using the VMD program and then analyzed using the AMBER22 CPPTRAJ package [42]. In particular, the RMSD was calculated on the backbone atoms of the protein using the *rmsd* command, while the *atomicfluct* command was used to calculate RMSF values on the Cα atoms. Salt bridges were analyzed using the *hbond* command, but only residues with a net charge of +1 or −1 were considered, and a maximum distance threshold of 4 Å was chosen. The hydrogen bonds were analyzed using the *hbond* command without any modification. The distance of the most relevant interactions was analyzed using the *distance* command. For the purposes of the analysis, interactions involving residues ≤ 4 positions apart, interactions formed between residues located on one of the two disordered loops, and those with a frequency < 10% of the total duration of the simulation were excluded from the results. Finally, the data were visualized and graphically represented using Python’s pandas [43] and matplotlib [44] libraries. The Cα RMSF values were mapped on the structural models using the same python libraries and visualized using ChimeraX 1.12 [45].

### 2.5. Clustering Procedure

To extract the most relevant conformations sampled by the systems during the molecular dynamics simulations, the *kmeans* algorithm from the CPPTRAJ package was used. The entire simulation trajectory was included in the analysis of all the simulations. Representative structures of the inward–open, occluded, and outward–open conformations, along with the G80S and N174I mutants, were obtained through this clustering procedure based on the RMSD values of the atoms of the main chain of the transmembrane helices. More precisely, residues 24–234 were chosen for the first 6 α helices (N–lobe) and residues 308–391 and 452–549 were selected for the last 6 α helices (C–lobe), thus excluding the two intra- and extracellular loops and the N- and C–terminal ends of the transporter from the procedure. The input number of clusters for the *kmeans* algorithm was set as a range from 2 to 20. At the end of the clustering procedure, the optimal number of clusters was determined using the elbow method by plotting the SSR/SST ratio against the number of clusters. The value corresponding to the ‘elbow’ of the graph was selected as a threshold beyond which adding extra clusters yields diminishing returns and may lead to overfitting.

The SSR/SST graphs for each MD replica are reported in Supplementary Figure S4.

## 3. Results

### 3.1. Validation of the hFPN Structural Models

The inward–open, occluded, and outward–open conformations of hFPN were obtained using the protein sequence of hFPN (Uniprot ID: Q9NP59) as an input for the ColabFold modeling software [32] (Figure 1, upper panels). The pLDDT and PAE ColabFold confidence metrics values for each hFPN structural model are reported in Supplementary Figure S2 and show an overall high confidence in the prediction of the transmembrane α–helices for each conformation. Furthermore, the pTM values for the inward–open, occluded, and outward–open conformation are, respectively, 0.74, 0.72, and 0.74. On the other hand, as expected, the pLDDT and PAE values indicate a lower confidence of the prediction of the unstructured regions corresponding to the ICL3 and ECL5, and the N-terminus and C-terminus of hFPN (Supplementary Figure S2).

To assess whether these ferroportin models correctly depicted the various conformations of the human iron exporter, we compared the arrangement of the transmembrane α–helices in the three conformational states with that of the helices from the experimentally determined structures of ferroportin and other MFS/SLC transporters available on PDB (Protein Data Bank) using the TM-align algorithm [37]. A comprehensive table of the results obtained from the comparison of ferroportin models reported in this work with the available experimental structures is shown in Supplementary Table S3.

For the inward–open conformation, since there are no experimentally determined structures of hFPN in this conformational state, the ColabFold model was compared with the *Bdellovibrio bacteriovorus* FPN (BbFPN) inward–open conformation (PDB code: 5AYO) (Figure 1, left) [8]. The alignment algorithm returned a TM-score of 0.9 and an RMSD (root mean square deviation) value of 2.44 Å.

For the ColabFold occluded and outward–open hFPN models, we compared them with their experimentally determined equivalents obtained via Cryo-EM [18,46].

The comparison between the occluded Cryo-EM structure and our structural model (Figure 1, middle) yielded a TM-score of 0.9 and an RMSD value of 2.95 Å. Interestingly, the experimentally obtained occluded conformation of hFPN with which we compared our model was isolated in complex with vamifeport (PDB code: 8BZY) [46], an oral inhibitor of ferroportin that is currently under clinical investigation for the treatment of sickle cell disease and β-thalassemia [47]. This compound has recently been suggested also to have a high potential as an adjunct therapy to treat lupus nephritis [48].

Finally, the structural model of the outward–open conformation was compared with the Cryo-EM structure of the same hFPN conformational state (PDB code: 6W4S) (Figure 1, right) [18] obtaining a TM-score of 0.96 and an RMSD value of 1.42 Å. Considering that a TM-score value between 0.5 and 1.0 indicates that the two proteins overall share the same fold [37], these results indicate that our predicted hFPN models of the inward–open, occluded, and outward–open conformations share high structural similarity with the corresponding human and/or bacterial homolog experimentally determined structures. Furthermore, each predicted hFPN model yielded TM-score values ≥ 0.7 when compared to the other MFS/SLC transporters (Supplementary Table S3) validating the use of these models for the subsequent molecular dynamics simulations studies (see Section 3.2).

### 3.2. Molecular Dynamics Simulations

To identify the residues involved in the differential stabilization of the three main conformations of ferroportin and the role of the residues that, when mutated, lead to the onset of ferroportin-related iron overload, the structural models of hFPN in the various conformational states were subjected to MD simulations. For each model of the three hFPN conformations, 1 µs MD simulations were carried out in three replicas. For the analysis of the intramolecular interactions, data obtained in a previous study carried out on the bacterial homolog of hFPN, the *Bdellovibrio bacteriovorus* BbFPN [49], were taken as a reference.

The known pathogenic mutations of hFPN are listed in Table 1 and have been mapped onto the inward–open, occluded, and outward–open conformations (Supplementary Figure S5).

#### 3.2.1. Global Mobility Analysis

The comparison between the RMSD values of the transmembrane α–helices in the various replicas of the wild-type systems (Supplementary Figure S6) indicates a general stability of all the three conformations, with RMSD values under 3.0 Å, except for the second replica of the inward–open conformation, the third replica of the occluded state, and the second replica of the outward–open conformation. On the other hand, the G80S mutant yields RMSD values between 3.0 and 4.0 Å, while the N174I graph reports similar values compared to the outward–open conformation replicas. The RMSF (root mean square fluctuation) values of the Cα atoms extracted from the various wild-type and mutant MD simulations highlight the presence of four particularly mobile areas in the three conformations of hFPN, corresponding to the two intracellular (ICL3) and extracellular (ECL5) loops, as well as the N–terminal and C–terminal ends (Supplementary Figure S7). This is in line with the fact that these regions are highly flexible and are not resolved in the available Cryo-EM experimental structures of hFPN [18]. To better evaluate how the mobility of the various transmembrane α–helices changes between the various conformations of hFPN, the coordinate files of the most representative structures from the various MD replicas of each system were extracted via a clustering procedure (for more details see the Section 2) and the Cα RMSF values were mapped onto the structural models values (Supplementary Figure S7).

It is not possible to state which α–helices are differentially more or less mobile in a specific conformation of hFPN based on the Cα RMSF values, as they change in an apparently incoherent way from one replica to another in the same hFPN conformational state (Table 2). Furthermore, the results indicate that, across the various MD simulations of hFPN, the mean value of the mobility of the transmembrane α–helices is similar between each conformation of the human iron exporter. Interestingly, as can be seen from Table 2, the RMSF values obtained from the MD replicas of the G80S and N174I mutants do not differ significantly from those of the wild-type replicas. This was surprising, especially for the G80S mutant whose pathogenic effect has been hypothesized to rely on the destabilization of the outward–open conformation [146]. Based on these results, we decided to focus on the wild-type systems for the analysis of the global hFPN stability (Figure 2) and, among the two mutant proteins, to take into account only the N174I mutant MD replicas for the subsequent intramolecular interactions analysis (see Section 3.2.2).

Overall, in all of the three conformations of hFPN, the α–helices apparently form a stable central core flanked by higher-mobility peripheral parts. More specifically, in each conformation, the RMSF values delineate a central core formed mainly by the stable α–helices TM1, TM5, TM7b, TM8, and TM10, while on the peripheral side of the protein, the remaining α–helices are more mobile, like TM6 and TM3 (Figure 2). Interestingly, the TM1, TM5, and TM7b α–helices are involved in iron binding and, with the exception of TM7b, they do not change their position significantly during the MD simulations. Moreover, TM8 is located closer to TM5 in the outward–open conformation, with respect to the occluded one.

Interestingly, in the outward–open conformation, TM7b is not only more mobile but it also changes its orientation with respect to that observed in the inward–open and occluded conformations, in which it is located near the TM1 α–helix that contains the S1 iron-binding site (D39–H43) (Figure 3). It can be hypothesized that this α–helix moves away from TM1 during the occluded–outward conformational change. The altered position of TM7b likely results from the flexibility of the small loop that connects TM7a to TM7b. On this loop is located a glycine residue (G323) that, when mutated to valine, causes the onset of ferroportin disease [64]. However, the role of G323 is still unclear [120]. Based on the results of the MD simulations of the three conformations, it appears that this residue allows TM7b to move away from the core of the transporter during the final phase of the iron transport (Figure 3).

#### 3.2.2. Intramolecular Interactions Analysis

For the analysis of the intramolecular interactions, the information available from previous MD simulations carried out on the experimental structures of the hFPN bacterial homolog BbFPN [147] was taken into consideration.

##### Electrostatic Interactions in Wild-Type hFPN

In each MD simulation of each conformation, two of the most stable salt bridges during the simulations are formed between R88 and D157, and between R40 and D181 (Figure 4). Interestingly, these two electrostatic interactions, and particularly, the R40–D181 one, are more persistent in the outward–open conformation than in the inward–open and occluded ones. R88–D157 is one of the interactions known to be responsible for the formation of the intracellular gate that stabilizes the outward–open conformation of hFPN [7]. For both R88 and D157, mutations have been documented (R88G and D157G) that result in reduced protein stability and loss of both cell surface localization and iron transport function [102]. On the other hand, the interaction between R40 and D181 represents the counterpart of the interaction R17–D162 that in the bacterial transporter stabilizes the inward–open conformation [147]. Interestingly, D181 is thought to be required for the initial phase of the iron transport in hFPN [6,148]. Furthermore, the D181V and R40Q mutations result in a marked and partial reduction of iron transport by hFPN, respectively, along with a more or less marked resistance to hepcidin [55,89].

As mentioned above, the D181 residue, which is located in the TM5 α–helix, is thought to participate in the initial phase of the iron transport mediated by hFPN [6]. Interestingly, in the occluded conformation, the α–helices TM5 and TM7b are close to each other, the TM7b hosting the S2 iron binding site (C326–H507) (Figure 5). In detail, the distances between D181 and D325 go from 12.8 ± 1.6 Å in the inward–open conformation to 11.5 ± 1.5 Å in the occluded one and finally to 13.5 ± 2.2 Å in the outward–open conformation. Analogously, D181 moves also towards R466, located in the TM11 α–helix, in the occluded and outward–open conformations (Figure 5). The proximity between TM5 and TM11 is expected since the intracellular gate that stabilizes the outward–open conformation involves the interaction between these two α–helices [18]. In the most representative structures extracted from the MD simulations of the wild-type systems, D181 and R466 are far from each other in the inward–open conformation (13.2 ± 1.4 Å) while they are closer in both the occluded and outward–open conformations (11.5 ± 1.6 Å and 10.7 ± 1.0 Å, respectively) (Figure 5). Furthermore, the R466M mutation has been demonstrated to disrupt iron transport in hFPN [6]. Interestingly, R466 is predicted to interact with D325 in the inward–open conformation (at a distance of 6.5 ± 1.6 Å), while it progressively moves away from this residue in the other two states. In fact, the distance between the two residues increases to 6.9 ± 1.4 Å in the occluded conformation and finally to 9.5 ± 1.6 Å in the outward–open one (Figure 5).

##### Hydrogen Bonds in Wild-Type hFPN

Regarding the hydrogen bonds formed during the MD simulations of wild-type hFPN, two of them involve at least one residue that leads to the onset of ferroportin-related iron overload when mutated. The first one is established between residues Y64 and N144, while the second one involves the N174 and Q478 residue couple (Figure 6). For the former interaction, it is interesting to note that both the gain-of-function mutations Y64N [61] and N144H [59] have been reported to lead to the onset of SLC40A1-HC, causing a reduction in the binding of hepcidin and in the subsequent ubiquitin-mediated degradation of hFPN [61]. The hydrogen bond formed by Y64 and N144, while being stable in the MD simulations of the outward–open conformation, with frequencies of occurrence near 37%, is nearly absent in both the occluded (frequency ~ 18%) and the inward–open simulation replicas (frequency ~ 12%). For the N174-Q478 hydrogen bond, the two residues involved are located in the TM5 and TM10 α–helices, which are known to establish interactions needed for the formation of the intracellular gate that stabilizes the outward–open conformation [7]. Furthermore, Q478 is thought to be essential for the protein stability and the conformational cycle [129], while the N174I mutation has been documented as responsible for ferroportin disease and associated with reduced iron transport and consequent hFPN dysfunction [6,7]. This hydrogen bond, not observed in the inward–open conformation and established only in the first replica of the occluded state MD simulations, is maintained for at least ~49% of the duration of the outward–open ones (Figure 6) Therefore, present MD data support the notion that this interaction contributes to the formation of the intracellular gate needed for the stabilization of the outward–open conformation [18].

Previously, the polar N174 residue was shown to be located in a strong hydrophobic environment in the outward–open conformation [6,18]. A coherent situation can be observed as well in the most representative structures from the MD simulations of the occluded and outward–open structural models. In detail, N174 in these two conformations is surrounded by residues I159, L170, I177, F360, and L474 (Figure 7, upper panels). We further analyze this issue in the following section.

##### Possible Pathogenic Mechanism for the N174I Mutation

Previously, N174I was hypothesized to lock hFPN in a non-functional outward–open state due to the formation of strong hydrophobic interactions with the surrounding microenvironment [6]. To verify this hypothesis, we carried out two MD simulation of 1 µs each of the N174I mutant in the outward–open conformation. While on one hand, the RMSD and RMSF analysis of both replicas revealed no significant differences between the mutant protein and the wild-type outward–open conformation (Supplementary Figures S6 and S7), on the other hand, the hydrogen bond between N174 and Q478 is obviously absent in each mutant replica and the mutated N174I residue establishes hydrophobic interactions for a substantial proportion of the trajectories with some of the residues found in the predicted hydrophobic environment. More in detail, the most stable one is formed with L474, while weaker hydrophobic interactions are also established with L170 and I159 (Figure 7, lower panels).

These results reinforce the notion that the N174I mutation traps the protein in the outward–open conformation by both disrupting the N174-Q478 hydrogen bond and promoting the formation of strong hydrophobic interactions with the surrounding environment, thus making the transporter unable to cycle back to the inward–open conformation.

## 4. Discussion

Ferroportin, or SLC40A1, is the only known iron exporter and specific missense mutations are associated with the onset of ferroportin-related iron overload. To this day, there are no experimentally determined structures of the inward–open conformation of hFPN and the Cryo-EM structures of the occluded and outward–open conformations do not include the two intracellular and extracellular loops, while both regions seem to be essential for the degradation of hFPN [4] and the final phase of the iron export [31], respectively. Given these data, the production of structural models of the whole hFPN protein structure in its conformations proved necessary to better understand the structural features and the dynamics of SLC40A1.

Regarding the hFPN structural models produced by ColabFold and described in this work, the pLDDT values for the transmembrane α–helices indicate a high/very high confidence, also in accordance with low PAE values (Supplementary Figure S2). The only regions whose prediction yields lower confidence scores are the two ICL3 and ECL5 regions of hFPN, along with the N–terminus and C–terminus. This is expected since these protein regions had to be removed due to their high flexibility in order to obtain experimental structures of hFPN [18].

As a confirmation of the reliability of our hFPN models in correctly representing the three main conformational states of the human iron exporter, the TM-score yielded values ≥ 0.86 when comparing the transmembrane α–helices of our models with those of experimentally determined structures of ferroportin. Furthermore, values between 0.74 and 0.8 were obtained when comparing them to other SLC/MFS transporters.

The subsequent MD analysis of the various hFPN systems yielded overall stable RMSD values for at least 2/3 replicas in both the wild-type systems and the mutated ones. The RMSF values for each residue are in accordance with the ColabFold pLDDT and PAE values, since the transmembrane α–helices share an overall stability in each conformation, while the two intracellular and extracellular loops, as well as the N–terminus and C–terminus of our three structural models, share higher RMSF values and therefore a higher mobility.

In every MD replica of each conformational state of wild-type hFPN, the iron exporter is characterized by the presence of a stable core mainly formed by TM1, TM5, TM7b, TM8, and TM10, surrounded by more mobile peripheral helices, such as TM6 and TM3. Of the helices making up the central core, TM7b and TM8 change their position from the inward– to the outward–open conformation. Interestingly, TM1 hosts the first iron binding site in hFPN, formed by the D39–H43 couple, while TM5 hosts the D181 residue that has been recognized to actively participate in the initial phase of iron export in hFPN [6,148]. Considering these results, it is tempting to speculate that TM1 and TM5 act like a “pivot” in this hypothetical core around which the other α–helices move and reorganize to complete the conformational transition and the iron export.

Next, we proceeded by examining how pathogenic LOF and GOF mutations on specific residues could impair the stability of hFPN and/or of its specific conformational states in order to understand how these alterations overall impair iron transport and result in ferroportin-related iron overload. The LOF mutations are responsible for the onset of ferroportin disease and are associated with an impairment in iron transport and/or membrane localization of hFPN; On the other hand, the GOF mutations lead to ferroportin-related hemochromatosis and mostly affect the binding of hepcidin to the iron exporter [25,26].

The LOF category comprises mutations such as G323V that was reported in a patient with ferroportin disease and unexplained hyperferritinemia [64]. This residue is part of the TM7b α–helix, which notably changes its position and orientation in the outward–open state, with respect to the inward–open and occluded ones. Considering the fact that G323 function in hFPN remains unclear to this day [120], we hypothesize that this glycine residue is essential for the conformational switch from the occluded to the outward–open conformation, since its intrinsic flexibility would allow the movement of TM7b away from the core of the transporter in the final phase of iron export. Therefore, the alteration of iron transport resulting from the G323V mutation likely derives from the lower flexibility of the loop connecting TM7a to TM7b and the impossibility of TM7b to move away from the core of the transporter at the end of the iron transport.

Notably, some LOF mutations not only impair the iron export of hFPN but also display a reduction in hepcidin binding to hFPN, making this distinction between LOF and GOF mutations reliable mainly by analyzing the patient’s altered clinical parameters and not the altered hFPN function.

For example, the D181V mutation has been reported in a case of ferroportin disease causing both an impairment of the iron export and hepcidin-resistance in hFPN [99]. Given the fact that D181 has been hypothesized to play a key role in the initial phase of iron export [6,148] and that during our MD simulations it establishes one of the two most stable electrostatic interactions in all of the three wild-type conformations with R40, especially in the outward–open one, we can hypothesize that the hepatic siderosis resulting from the D181V mutation primarily results from a perturbation of the overall stability of hFPN, causing an impairment of both the iron export and the binding of hepcidin. Moreover, D181 is located near R466 in the TM11 α–helix in both the occluded and outward–open conformation, while this basic residue moves away from D325 in the TM7b α–helix from the occluded to the outward–open state. Given the fact that D181 is also more mobile in the inward–open and in the occluded states, one possibility is that iron, once inside hFPN, could be “guided” towards the S2 binding site by the movement of TM5 while tethered to D181 via electrostatic interactions. In agreement with this hypothesis, it has been suggested that D181, along with the residues of the S1 iron binding site in TM1, i.e., the couple D39–H43, participate in the initial phase of iron transport [6,148]. Considering these results and both the fact that R466 changes its binding partner between the three main conformations of hFPN and that D325 has been demonstrated to be essential for iron transport [149], one of the possible mechanisms that emerges is that Fe^2+^ could be initially recruited by an ion binding site made up by D181 and the D39–H43 couple in the inward–open conformation; subsequently, D325 would break the interaction with R466 to bind the metal, leaving this basic residue free to relocate near D181 in order to facilitate the inward–outward conformational transition and the concomitant iron export from hFPN. However, additional experimental structural data and advanced MD simulations able to explore conformational transitions, such as enhanced sampling techniques, are needed to verify or disprove this hypothetical mechanism.

Ferroportin disease results also from the D157G LOF mutation. This aspartate residue has been observed to establish a stable salt bridge with R88 during all our MD simulations. Similarly, R88 was also reported in a case of ferroportin disease when mutated to glycine and together with D157G both mutations cause an altered protein stability and, in the case of D157G, a mislocalization in the cytoplasm of hFPN [102]. Similarly to D181V, the hepatic iron overload phenotype observed in patients with one of these mutations likely results from the loss of this salt bridge that would impair the correct stability and folding of this mutant form of hFPN.

Finally, the N174 residue was shown to cause the ferroportin disease phenotype when mutated to isoleucine, leading to liver and intestinal iron accumulation and a reduction in iron export by hFPN [7,94]. This mutation was hypothesized to lock hFPN in a non-functional state [6]. Based on these results, this LOF mutation could cause this pathogenic phenotype for two possible reasons: the first one is the breakage of a hydrogen bond in the outward–open conformation between N174 and Q478 that would keep ferroportin in the inward/occluded conformation and consequentially impair the iron export mechanism. The second reason could be the fact that N174, when mutated to isoleucine, forms strong hydrophobic interactions with surrounding residues such as L474, L170, I159, and F360. Interestingly, the latter hypothesis is confirmed by the MD simulations of the outward–open conformation of N174I FPN, since in these simulations, the I174 residue establishes hydrophobic interactions with three of the hypothesized residues, i.e., L474, I159, and L170. Thus, it appears realistic that the altered intra-protein environment could block hFPN in the outward–open state and prevent the reversion from this conformation to the inward–open one.

It is interesting to notice that these LOF residues are located at the inter–lobes interface (Supplementary Figure S5), a feature that was already observed for the orthologous residues of the bacterial homolog of hFPN [8].

On the other hand, the majority of the GOF mutations, as previously noted in [146], are located in the vicinity of TM2 and TM11, where the binding site of hepcidin is found (Supplementary Figure S5) [18].

The GOF mutations Y64N and N144H are reported in cases of SLC40A1-HC with hepatic fibrosis and parenchymal iron overload [59,61]. Interestingly, the two wild-type residues form a hydrogen bond with each other in the MD simulations of the occluded conformation that becomes more stable in the outward–open state. Since the corresponding mutations lead to a reduction in the ubiquitin-dependent hFPN degradation, notwithstanding the binding of hepcidin, it can be hypothesized that the interruption of this interaction due to these mutations leads to an altered inward−outward equilibrium thereby making the protein less responsive to the hepcidin-induced degradation [50].

## 5. Conclusions

The production of in silico structural models of hFPN allowed us to elucidate the structural and dynamic features that alternatively stabilize the inward–open, occluded, and outward–open conformations. More in detail, we showed that AI-based prediction tools like ColabFold were able to produce reliable structural models of the three main conformations of hFPN, also with the protein regions that are unresolved in the experimentally obtained structures of the iron exporter, i.e., the two intracellular and extracellular loops and the N– and C–terminal ends.

We performed 1 µs MD simulations for each one of these models in three replicas to analyze which transmembrane α–helices of the iron exporter are actively or passively involved in the stabilization of the various conformations. We noticed that for each hFPN conformational state, the transmembrane α–helices form a central stable core flanked by more mobile peripheric regions and that helices like TM1 and TM5 may act like a “pivot” around which the other α–helices move to make the transporter alternatively more accessible to the intracellular or the extracellular milieu. On the other hand, α–helices such as TM7b probably change their orientation to finalize the iron export, and this flexibility could derive from the presence of a glycine residue at the N–terminal of this helix, namely, G323. Therefore, the presence of a bulkier residue in that position, like for the G323V pathogenic mutation, verisimilarly hinders the mobility of this protein region and the final phase of iron export.

Finally, we analyzed how specific intramolecular interactions such as salt bridges and/or hydrogen bonds can stabilize hFPN and/or its specific conformations in a physiological context and how their breakage could be responsible for pathological consequences in patients with ferroportin-related diseases. Based on our results, we hypothesize that pathogenic mutations such as R40Q, R88G, D157G, and D181V could lead to an incorrect protein folding due to the interruption of very stable electrostatic interactions established in each conformation; on the other hand, mutations like Y64N, N144H, and N174I could alter the stability of specific conformational states and therefore hFPN function. More in detail, the N174I mutation likely blocks hFPN in a non-functional outward–open state due to the strong hydrophobic interactions that it establishes within its microenvironment and to the breakage of a hydrogen bond persistently maintained in the outward–open conformation, i.e., the one between N174 and Q478. These two pathogenic mechanisms could, for example, impede the reversion from the outward–open to the inward–open conformation of hFPN after the binding of hepcidin, therefore jeopardizing the post-translational regulation of the iron exporter and leading to a general dysregulation in the intracellular iron homeostasis.

In conclusion, this work provides new in silico data on the inward–open, occluded, and outward–open conformations of the complete hFPN structure and of two of its pathogenic mutants that may provide useful insights into the dynamics of the transporter and the pathogenic role of certain mutations known to cause ferroportin-related iron overload. It must be stressed that in order to reliably simulate how specific mutations disrupt the conformational transitions of hFPN, we would need to approach this system with more advanced, computationally demanding, molecular dynamics techniques such as enhanced sampling. We plan to apply this approach in a natural follow-up of this work since this understanding may also motivate future studies aimed at developing new therapies for ferroportin-related iron overload capable of restoring the transport capacity of pathogenic hFPN mutants.

## Figures and Tables

**Figure 1:**
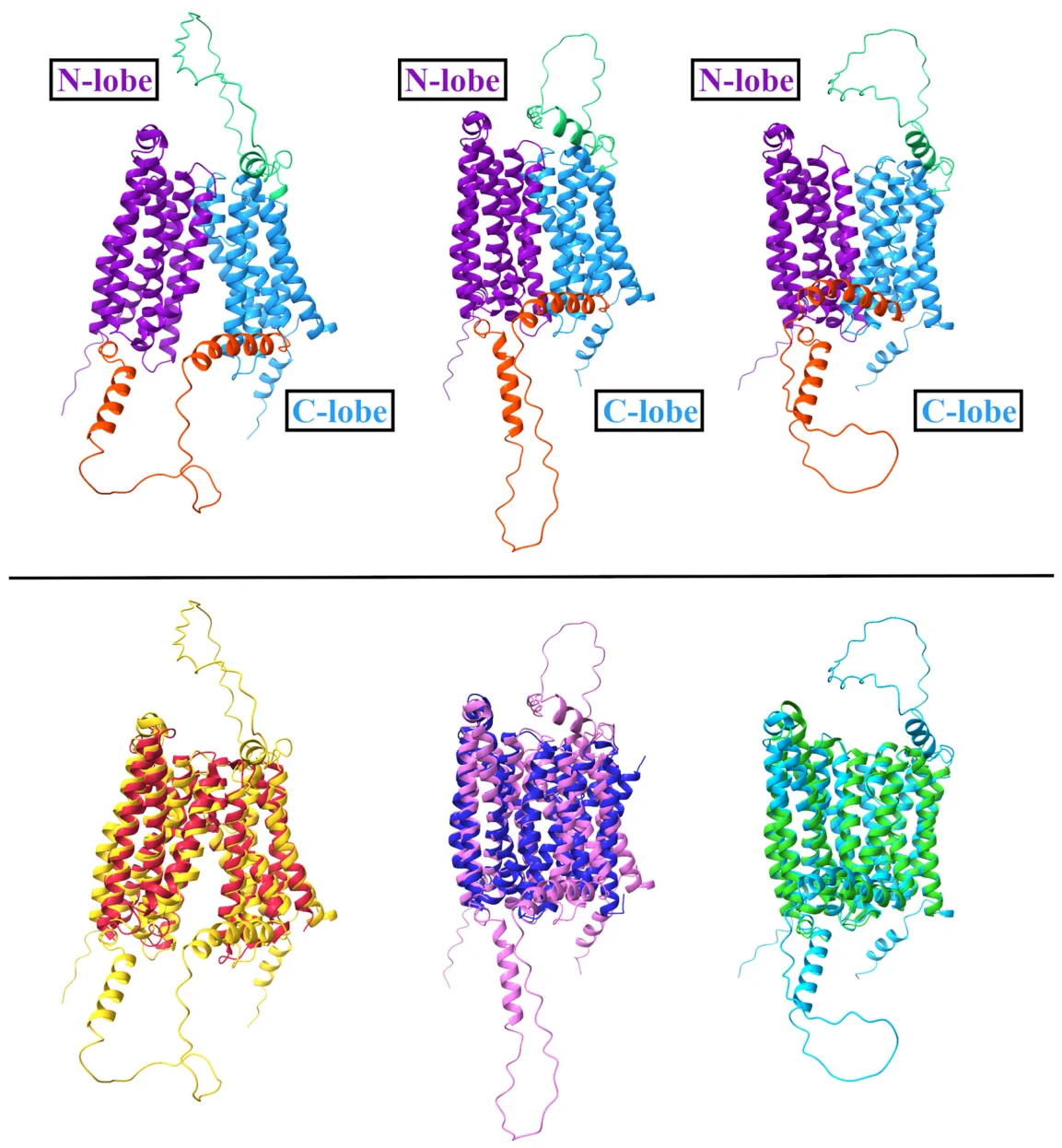
((**Upper**) panels) Colabfold structural models of human ferroportin in its inward–open (**left**), occluded (**middle**), and outward–open conformations (**right**), with the N–lobe and the C–lobe marked in purple and cyan-blue in the various conformations. The two intracellular and extracellular loops are colored in red-orange and green-cyan, respectively. ((**Lower**) panels) Comparison between the same computational structural models of the inward–open ((**left**), ochre), occluded ((**middle**), orchid), and outward–open ((**right**), deep blue sky) conformations with the experimental structures of the BbFPN inward–open ((**left**), red), hFPN occluded ((**middle**), dark blue), and hFPN outward–open ((**right**), green) conformations. For details see text.

**Figure 2:**
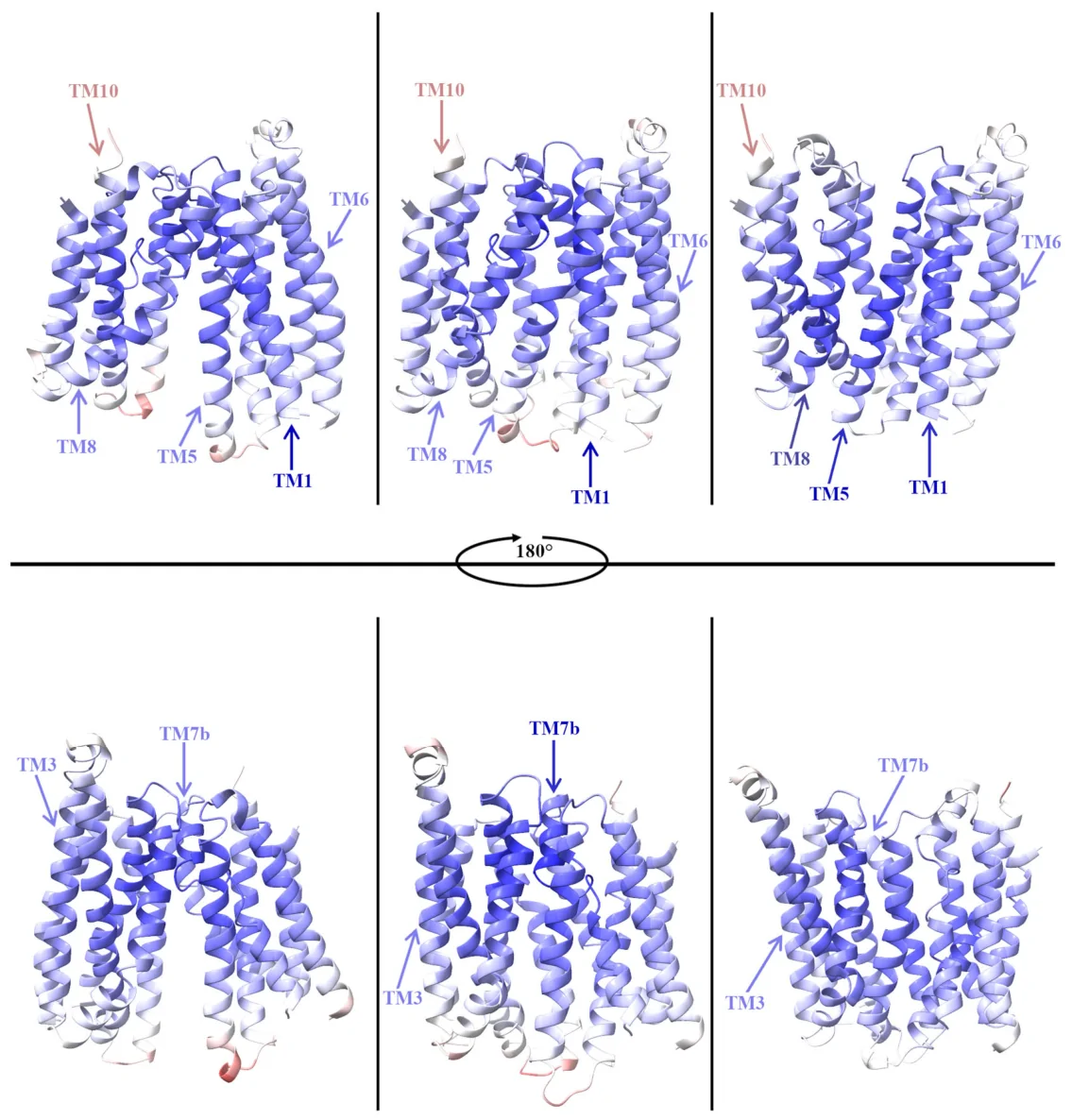
RMSF mean values from the MD simulations of each wild-type hFPN conformation mapped onto the structure of the centroid of the most populated cluster of the first MD replica of that specific state, seen from a frontal ((**upper**) panels) and a posterior view ((**lower**) panels): from the left, centroid of the MD simulation of the inward–open ((**left**), (**upper**), and (**lower**) panel), occluded ((**middle**), (**upper**), and (**lower**) panel) and outward–open conformation ((**right**), (**upper**), and (**lower**) panel). In each structure, blue corresponds to lower RMSF values while red to higher RMSF values.

**Figure 3:**
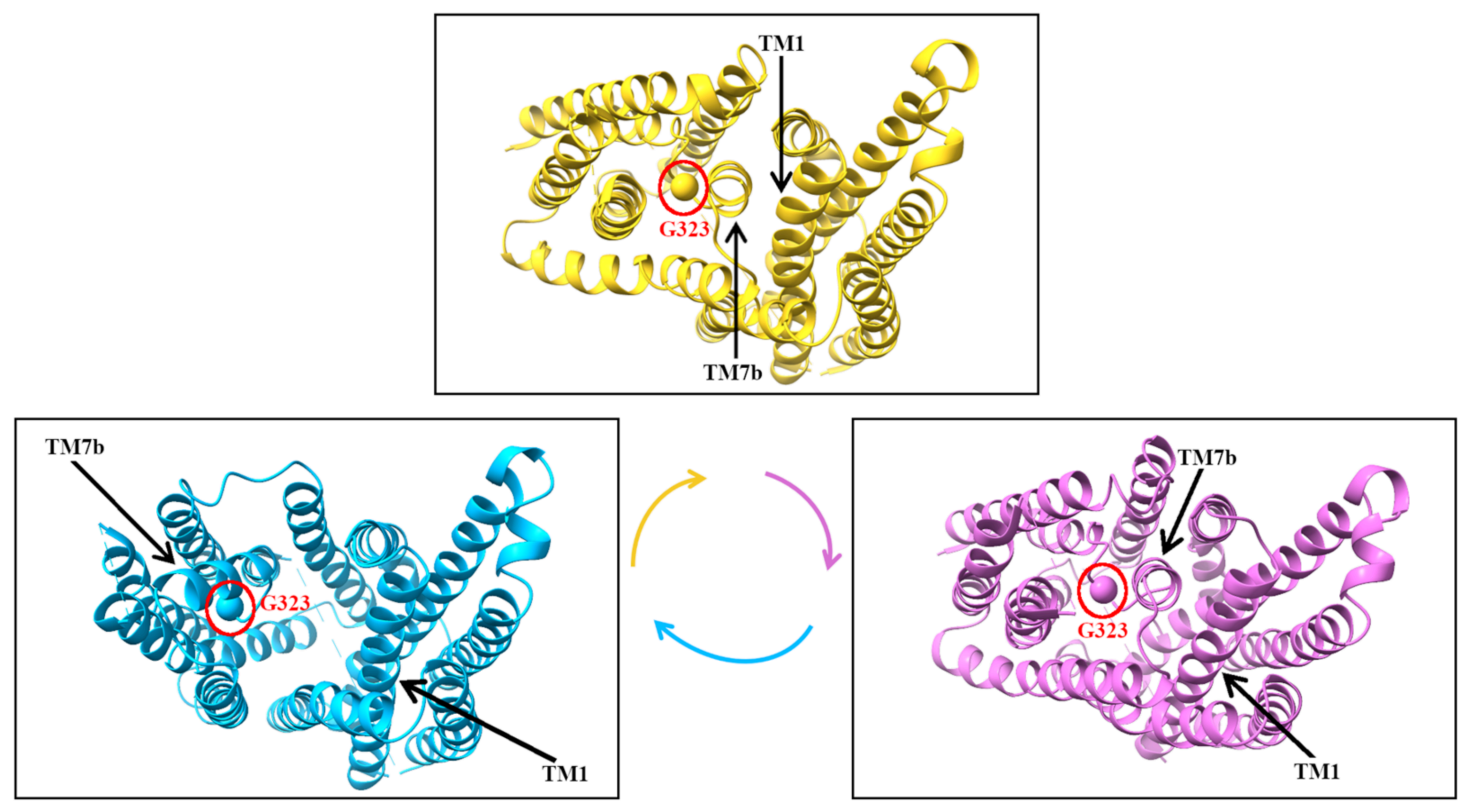
Representation of the relative position of TM7b with respect to TM1 and of the G323 (sphere circled in red) residue in the three conformational states of hFPN. Inward–open conformation (on (**top**), yellow), occluded conformation (down on the (**right**), orchid), and outward–open conformation (down on the (**left**), deep sky blue). Curved arrows indicate the direction of the conformational changes from the inward–open to the outward–open conformation.

**Figure 4:**
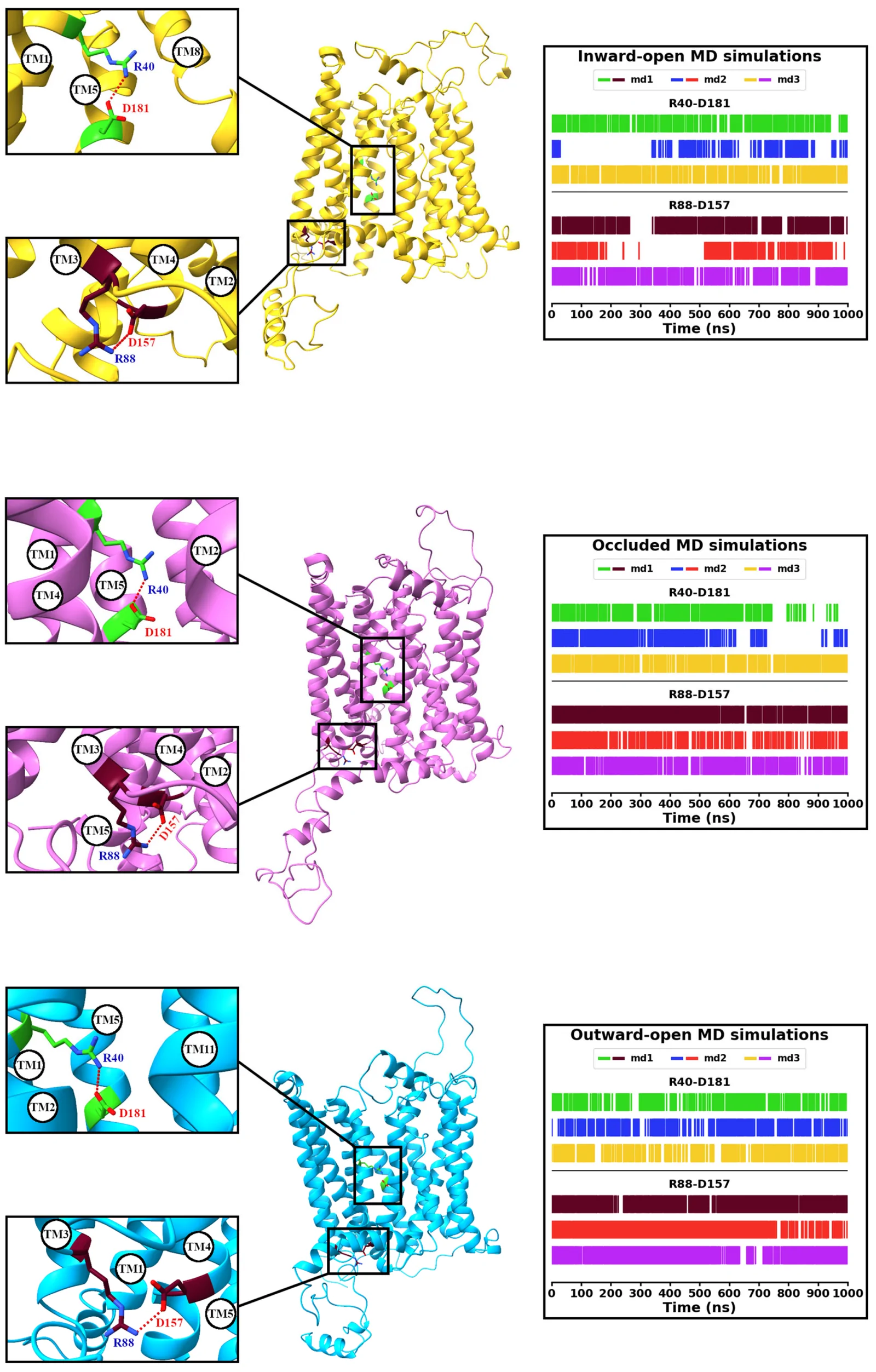
Representation of the most stable salt bridges observed in MD simulations of the inward–open ((**upper**) panel), occluded ((**middle**) panel), and outward–open ((**lower**) panel) conformational models of hFPN. ((**Left**) and (**middle**) panels) focus on the position of the residues forming the two interactions and visual localization of the same residues in the whole structure of the most representative frame of the first MD replica of each hFPN conformation. ((**Right**) panels) event plot graph showing the time series of the same interactions. The graph displays the frames in which each interaction was observed during the various MD simulations.

**Figure 5:**
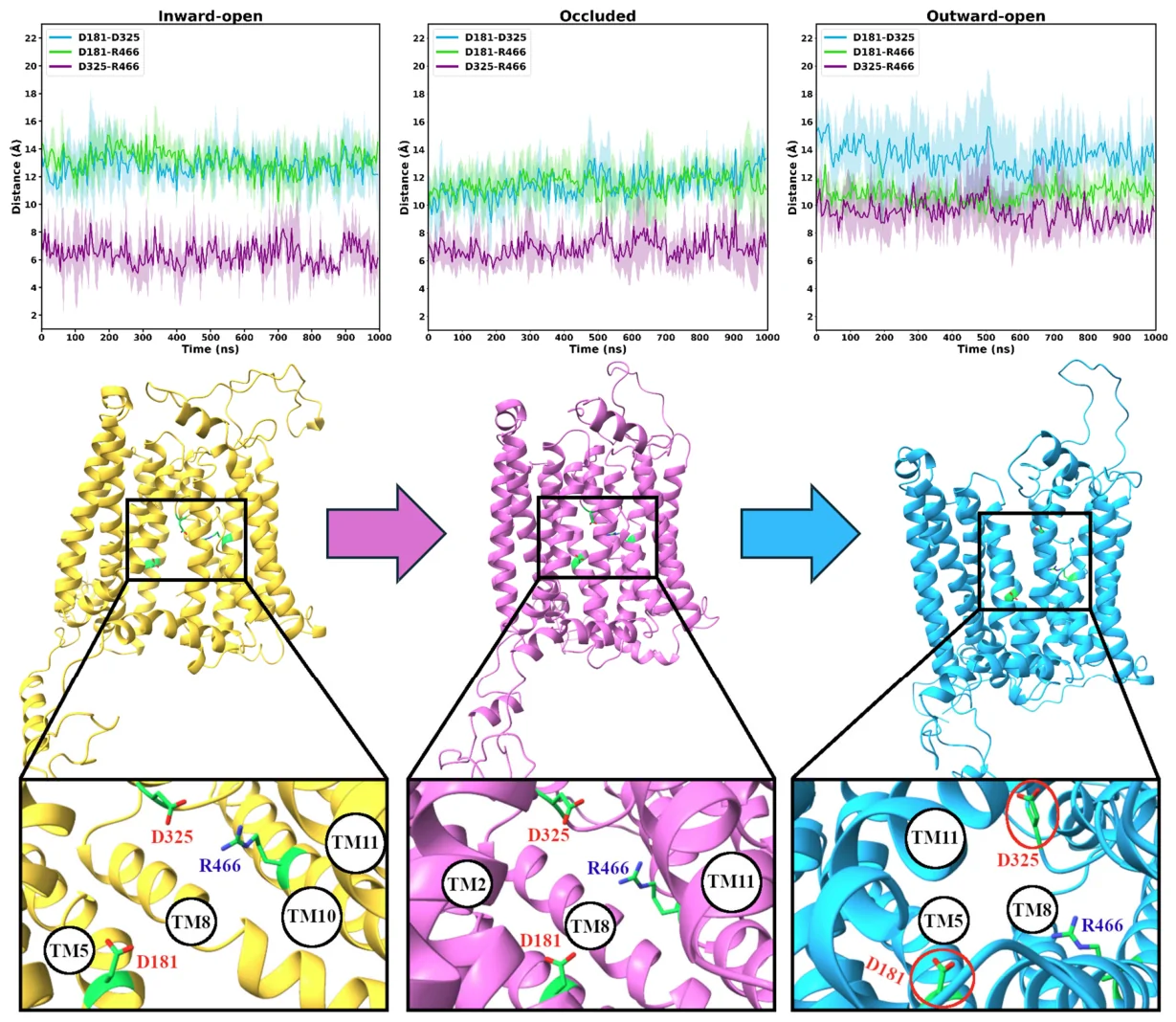
Representation of the relative position of D181, D325, and R466 in the three conformational states of hFPN. ((**Upper**) panels) average distances profiles between D181–D325 (light blue line), D181–R466 (green line), and D325–R466 (purple line) during the MD simulations of each conformation. Solid lines represent the mean values; shaded areas represent ± SD. ((**Lower**) panels) most representative structure of the first MD replica of the inward–open conformation ((**left**), yellow), occluded conformation ((**middle**), orchid), and outward–open conformation ((**right**) panel, deep sky blue). For each conformation, the location of the three residues in the whole structure of hFPN is shown on top, while the bottom panels show a focus of their structural environment.

**Figure 6:**
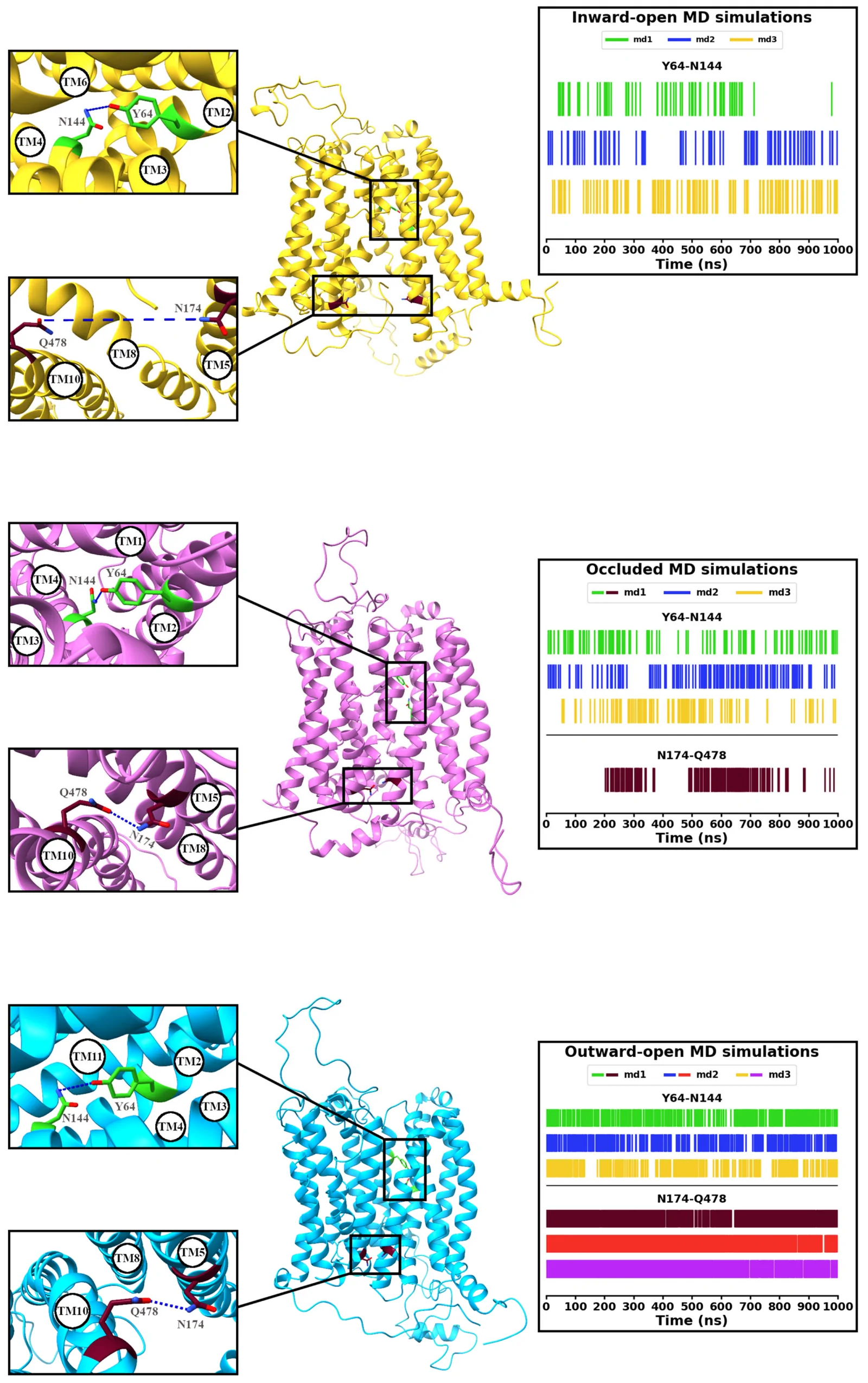
Representation of the Y64–N144 and N174–Q478 hydrogen bonds in the inward–open ((**upper**) panel), occluded ((**middle**) panel) and outward–open conformational models of hFPN. ((**Left**) and (**middle**) panels) focus on the position of the residues forming the two interactions and visual localization of the same residues in the most representative structure of the first MD replica of each hFPN conformation. ((**Right**) panels) eventplot showing the time series of the same interactions. The graph displays the frames in which each interaction was observed during the various MD simulations. The N174–Q478 hydrogen bond in the inward–open conformation was excluded from the event plot due to its total absence in each MD simulation of this conformational state, while in the occluded conformation, it is established only in the first replica.

**Figure 7:**
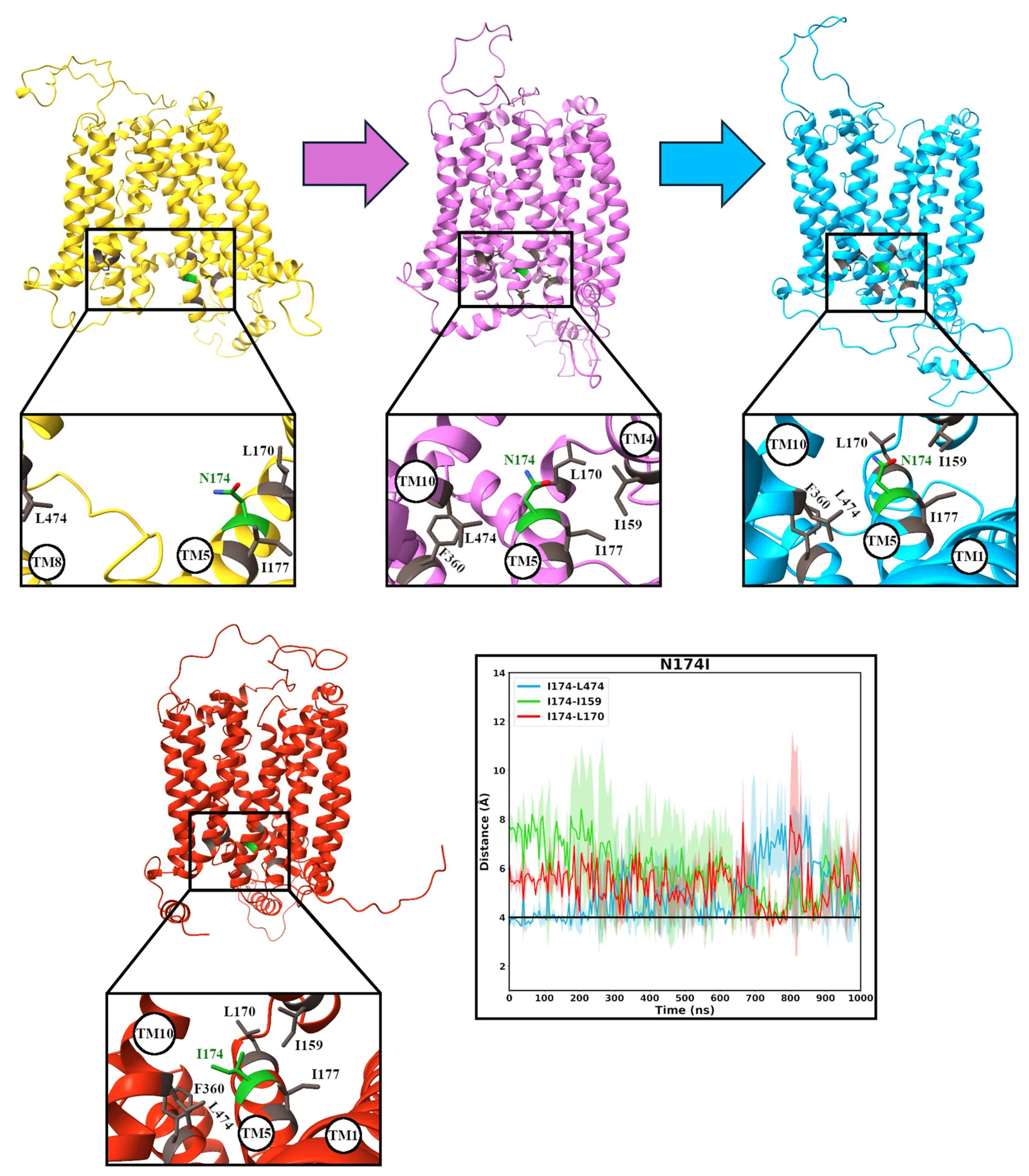
((**Upper**) panels) Movement of N174 in an increasingly hydrophobic environment going from the inward– to the outward–open conformation of hFPN. Inward–open ((**left**), yellow), occluded (**middle**), orchid), and outward–open conformations ((**right**), deep sky blue). For each conformation, the localization of the various residues in the whole structure of hFPN is shown on top, while on the bottom, there is a focus on their structural environment. ((**Lower**) panel) I174 is surrounded by a hydrophobic environment that likely blocks human ferroportin in a non-functional outward–open state (on the (**left**)). (On the (**right**)) average distances profiles between I174–L474 (light blue line), I174–I159 (green line), and I174–L170 (red line) during the MD simulations of the N174I pathogenic mutant. Solid lines represent the mean distance; shaded areas represent ± SD. Only the interactions that fall below the 4 Å threshold are plotted.

**Table 1:** Comprehensive annotation of all the known mutations of hFPN, classified on the basis of their identification in patients with SLC40A1-related hemochromatosis (SLC40A1-HC) or with ferroportin disease (FD), or else if their pathogenic effect is null (Null) or unknown (Uncertain significance). The known pathogenic mutations in SLC40A1-HC and FD are also sub-classified as hepcidin-resistant (HAMP-r), hepcidin-sensitive (HAMP-s), and hepcidin-uncertain (HAMP-u).

Pathogenic Variant	HGVS Nomenclature	In Vitro Hepcidin Response	References
SLC40A1-related hemochromatosis (elevated TSAT and elevated ferritin levels)			
V63I	p.Val63Ile	HAMP-u	[49]
Y64N	p.Tyr64Asn	HAMP-r	[50,51,52]
Y64H	p.Tyr64His	HAMP-u	[53]
A69T	p.Ala69Thr	HAMP-u	[54,55]
V72F	p.Val72Phe	HAMP-u	[50,56]
N144D	p.Asn144Asp	HAMP-u	[50,52,57,58]
N144H	p.Asn144His	HAMP-u	[52,58,59,60]
N144T	p.Asn144Thr	mild HAMP-r	[60,61,62]
Q182H	p.Gln182His	HAMP-u	[63,64,65,66]
N185D	p.Asn185Asp	HAMP-s	[67,68,69]
G204S	p.Gly204Ser	HAMP-u	[50,67,68,70]
G204R	p.Gly204Arg	HAMP-u	[54]
K240E	p.Lys240Glu	HAMP-r	[71,72]
D270V	p.Asp270Val	mild HAMP-r	[50,73,74]
C326F	p.Cys326Phe	HAMP-u	[75,76,77]
C326S	p.Cys326Ser	HAMP-r	[50,61,78,79]
C326Y	p.Cys326Tyr	HAMP-r	[22,52,61,80]
Y333H	p.Tyr333His	HAMP-r	[81]
S338R	p.Ser338Arg	HAMP-u	[50,82]
Y501C	p.Tyr501Cys	HAMP-r	[6,50,83]
D504N	p.Asp504Asn	HAMP-r	[50,84]
H507R	p.His507Arg	HAMP-r	[50,85,86,87]
V511I	p.Val511Ile	HAMP-u	[81,88]
Ferroportin disease (normal TSAT and elevated ferritin levels)			
R40Q	p.Arg40Gln	mild HAMP-r	[89]
S47F	p.Ser47Phe	mild HAMP-r	[89]
A77D	p.Ala77Asp	HAMP-u	[52,58,63,84,90,91,92,93]
G80S	p.Gly80Ser	HAMP-u	[84,92,94,95,96,97,98]
G80V	p.Gly80Val	HAMP-u	[99]
D84E	p.Asp84Glu	HAMP-r	[100]
R88G	p.Arg88Gly	HAMP-s	[68,84,101,102]
R88I	p.Arg88Ile	HAMP-u	[103]
L129P	p.Leu129Pro	HAMP-s	[104]
I152F	p.Ile152Phe	HAMP-u	[22,105]
D157G	p.Asp157Gly	HAMP-u	[63,64,65,66,84,102]
D157N	p.Asp157Asn	HAMP-u	[54,56]
D157Y	p.Asp157Tyr	HAMP-u	[84,106]
W158C	p.Trp158Cys	HAMP-u	[87,107]
W158L	p.Trp158Leu	HAMP-u	[68]
V162del	p.Val162del	HAMP-r	[58,60,63,65,66,84,91,96,108,109,110,111,112,113,114,115,116,117,118,119]
N174I	p.Asn174Ile	HAMP-u	[6,22,94,120]
R178Q	p.Arg178Gln	HAMP-u	[93,101,114,121]
D181N	p.Asp181Asn	HAMP-r	[54]
D181V	p.Asp181Val	HAMP-r	[6,55,84,99]
S209L	p.Ser209Leu	HAMP-u	[28,107,122]
L233P	p.Leu233Pro	HAMP-u	[84,105]
L233V	p.Leu233Val	HAMP-u	[123]
G267D	p.Gly267Asp	HAMP-u	[68]
G323V	p.Gly323Val	HAMP-u	[63,64,65,66,120]
A350V	p.Ala350Val	HAMP-r	[89]
T419I	p.Thr419Ile	HAMP-u	[124]
G468S	p.Gly468Ser	HAMP-u	[125,126]
R489K	p.Arg489Lys	HAMP-u	[127]
R489S	p.Arg489Ser	HAMP-u	[106,128,129,130,131]
G490D	p.Gly490Asp	HAMP-r	[58,66,84,120,132]
Null			
I180T	p.Ile180Thr	—	[84,133]
T230N	p.Thr230Asn	—	[84]
M266T	p.Met266Thr	—	[84]
L345F	p.Leu345Phe	—	[84]
I351V	p.Ile351Val	—	[84]
P443L	p.Pro443Leu	—	[84]
Uncertain significance			
A45E	p.Ala45Glu	—	[28]
S71F	p.Ser71Phe	—	[28,67]
R88T	p.Arg88Thr	—	[133]
D157A	p.Asp157Ala	—	[134,135,136]
V160A	p.Val160Ala	—	[137]
M173V	p.Met173Val	—	[28]
A175D	p.Ala175Asp	—	[28]
R178G	p.Arg178Gly	—	[28,138]
R179T	p.Arg179Thr	—	[137,139]
Q182R	p.Gln182Arg	—	[28]
N185T	p.Asn185Thr	—	[67]
G204V	p.Gly204Val	—	[28]
A232D	p.Ala232Asp	—	[140]
A237V	p.Ala237Val	—	[28]
Q248H	p.Gln248His	—	[52,58,84,141,142,143]
R296Q	p.Arg296Gln	—	[26,54]
A350D	p.Ala350Asp	—	[137]
R371Q	p.Arg371Gln	—	[67,68]
R371W	p.Arg371Trp	—	[67]
G490S	p.Gly490Ser	—	[84,101]
G494D	p.Gly494Asp	—	[137]
V531A	p.Val531Ala	—	[137]
R561G	p.Arg561Gly	—	[84,144,145]

**Table 2:** Average RMSF values of the transmembrane α–helices in each MD replica (second row) and in each conformation of hFPN (third row).

Conformation/Variant	Replicas (n)	Average RMSF/Replica (Å)	(Mean RMSF ± SD)
Inward–open	3	2.0, 2.2, 1.4	1.9 ± 0.4
Occluded	3	1.8, 1.7, 2.2	1.9 ± 0.3
Outward–open	3	2.2, 1.6, 1.8	1.9 ± 0.3
G80S	1	2.0	2.0
N174I	2	1.9, 1.7	1.8 ± 0.1

## Data Availability

The data that support the findings of this study are openly available in Zenodo at https://zenodo.org/records/22753365 (accessed on 17 September 2026); doi: 10.5281/zenodo.22753365; reference number 22753365.

## Supplementary Materials

The following supporting information can be downloaded at: https://www.mdpi.com/article/10.3390/biom16101382/s1, Table S1. List of the SLC40A1 homologs sequences chosen to perform the MSA with hFPN, Figure S1. Multiple sequence alignment of SLC40A1 homologs in the Animalia and Fungi kingdoms. Organisms included in the range delimited by the two solid black lines are the most representatives of the Vertebrates subphylum, Figure S2. Representation of the ColabFold pLDDT and PAE metric values plotted for each hFPN conformational model, Table S2. Quantity and proportions of lipids used to construct the plasma membrane in simulations of hFPN structural models, Figure S3. Representation of the initial structures of each hFPN conformation inserted in their own plasma membrane model: from the left, inward–open (yellow), occluded (orchid) and outward–open conformations (deep sky blue), Figure S4. Representation of the SSR/SST value (Y axis) plotted against the number of clusters (X axis) for each MD simulation of each hFPN system, Table S3. TM-align results from the comparison of our hFPN structural models with the experimentally determined structures of Ferroportin homologs and other MFS/SLC transporters, Figure S5. Representation of the three structural models of the inward–open (left), occluded (middle), and outward–open (right) conformations of hFPN. The spheres represent the pathogenic mutations known to cause Ferroportin–related iron overload, distinguished between “loss–of–function” (red spheres), “gain–of–function” (blue spheres), neutral (green spheres) and of uncertain significance (black spheres), Figure S6. Backbone RMSD values calculated on the three replicas of the MD trajectories of each of the analysed systems, Figure S7. Representation of the mean RMSF values with standard deviation from the MD simulations of the analysed hFPN wild–type and mutant systems from a graphical (left) and from a structural point of view (right).
