# Supplementary material for: Computational Studies of the Dynamics of the Human Iron Exporter Ferroportin and Insight into the Role of Crucial Residues Involved in Pathogenic Mutations
Source: Biomolecules. 2026 Sep 22;16(10):1382. doi: 10.3390/biom16101382 (PMC13702422; doi:10.3390/biom16101382)
Supplement: Supplementary file 1 [file biomolecules-16-01382-s001.zip › biomolecules-4505294-supplementary.pdf]

Supplementary Materials

Computational Studies of the Dynamics of the Human Iron Exporter Ferroportin and Insight into the Role of Crucial Residues Involved in Pathogenic Mutations

Sergio Vulterini [1], Maria Carmela Bonaccorsi di Patti [2], Giovanni Musci [3] and Fabio Polticelli [1,*]

[1] Department of Sciences, Roma Tre University, 00146 Rome, Italy; sergio.vulterini@uniroma3.it
[2] Department of Biochemical Sciences 'A. Rossi Fanelli', Sapienza University of Rome, 00185 Rome, Italy; mariacarmela.bonaccorsi@uniroma1.it
[3] Department of Biosciences and Territory, University of Molise, 86090 Pesche, Italy; giovanni.musci@unimol.it
* Correspondence: fabio.polticelli@uniroma3.it; Tel.: +39-0657336362

Table S1. List of the SLC40A1 homologs sequences chosen to perform the MSA with hFPN.

**Kingdom**	**Phylum (and subphylum)**	**Species**	**Uniprot ID**	**Protein name**
Fungi	Zoopagomycota	Basidiobolus *meristosporus CBS 931.73*	*A0A1Y1Y3J8*	Solute carrier family 40 member
Metazoa	Porifera	Corticium *candelabrum*	*UPI002E252686*	solute carrier family 40 member 1-like
Metazoa	Cnidaria	Rhopilema *esculentum*	*UPI0031DC3D0D*	uncharacterized protein LOC135681058
Metazoa	Ctenophora	Bolinopsis *microptera*	*UPI003079D07C*	solute carrier family 40 member 1-like
Metazoa	Platyhelminthes	Macrostomum *lignano*	*A0A1I8G572*	Solute carrier family 40 member
Metazoa	Nematoda	Toxocara *canis*	*A0A0B2W3X9*	solute carrier family 40 member
Metazoa	Nematomorpha	Gordionus *sp. m RMFG-2023*	*UPI0030E0D8E4*	solute carrier family 40 protein member 1-like isoform X1
Metazoa	Rotifera	Rotaria *sp. Silwood1*	*A0A813T9T6*	unnamed protein product
Metazoa	Tardigrada	Paramacrobiotus *metropolitanus*	*UPI0024455FB6*	uncharacterized protein F54H12.2-like
Metazoa	Arthropoda (Chelicerata)	Limulus *polyphemus*	*UPI0006B0E25E*	solute carrier family 40 member 1-like
Metazoa	Arthropoda (Chelicerata)	Galendromus *occidentalis*	*A0AAJ7WIA3*	Solute carrier family 40 member
Metazoa	Arthropoda (Crustacea)	Daphnia *sinensis*	*A0AAD5PW72*	Solute carrier family 40 member
Metazoa	Arthropoda (Hexapoda)	Timema *poppense*	*A0A7R9CJM0*	Solute carrier family 40 member
Metazoa	Mollusca	Mya *arenaria*	*UPI0022E0E3AB*	solute carrier family 40 member 1-like
Metazoa	Annelida	Capitella *teleta*	*R7UP72*	Solute carrier family 40 member
Metazoa	Bryozoa	Watersipora *subatra*	*UPI00355C9393*	solute carrier family 40 member 1-like
Metazoa	Echinodermata	Anneissia *japonica*	*UPI001425591D*	solute carrier family 40 member 1-like
Metazoa	Hemichordata	Ptychodera *flava*	*UPI00396A25DC*	solute carrier family 40 member 1-like
Metazoa	Chordata (Tunicata)	Phallusia *mammillata*	*A0A6F9DTD9*	Solute carrier family 40 member
Metazoa	Chordata (Cephalochordata)	Branchiostoma *belcheri*	*A0A6P4YLL3*	Solute carrier family 40 member
Metazoa	Chordata (Craniata)	Petromyzon *marinus*	*A0AAJ7SWZ6*	Solute carrier family 40 member
Metazoa	Chordata (Craniata)	Heptranchias *perlo*	*UPI00355A05D0*	solute carrier family 40 member 1
Metazoa	Chordata (Craniata)	Danio *rerio*	*Q9I9R3*	solute carrier family 40 member 1
Metazoa	Chordata (Craniata)	Polyodon *spathula*	*UPI001B7EB371*	solute carrier family 40 member 1 isoform X2

Metaz oa	Chordata (Craniata)	Rhinatrema *bivittatum*	*UPI001128 1C2D*	solute carrier family 40 member 1 isoform X1
Metaz oa	Chordata (Craniata)	Mauremys *mutica*	*UPI001D15 3B9F*	solute carrier family 40 member 1
Metaz oa	Chordata (Craniata)	Struthio *camelus australis*	*A0A093H4I 1*	Solute carrier family 40 member
Metaz oa	Chordata (Craniata)	Mus *musculus*	*Q9JHI9*	Ferroportin

= Residues required for iron binding

= Residues forming the "A motif"

= Residues required for calcium binding

= Residues involved in hepcidin binding

= Residues involved in the stabilization of the outward-open conformation

= Residues which interact with the membrane lipids

= Residues involved in the iron export

= Residues involved in the ubiquitination of hFPN

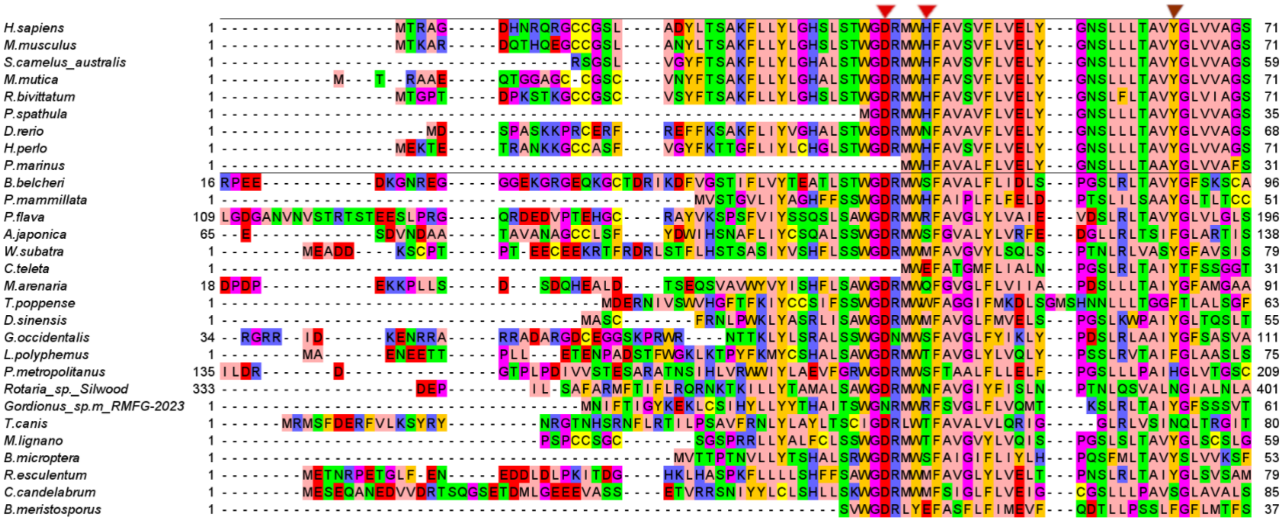

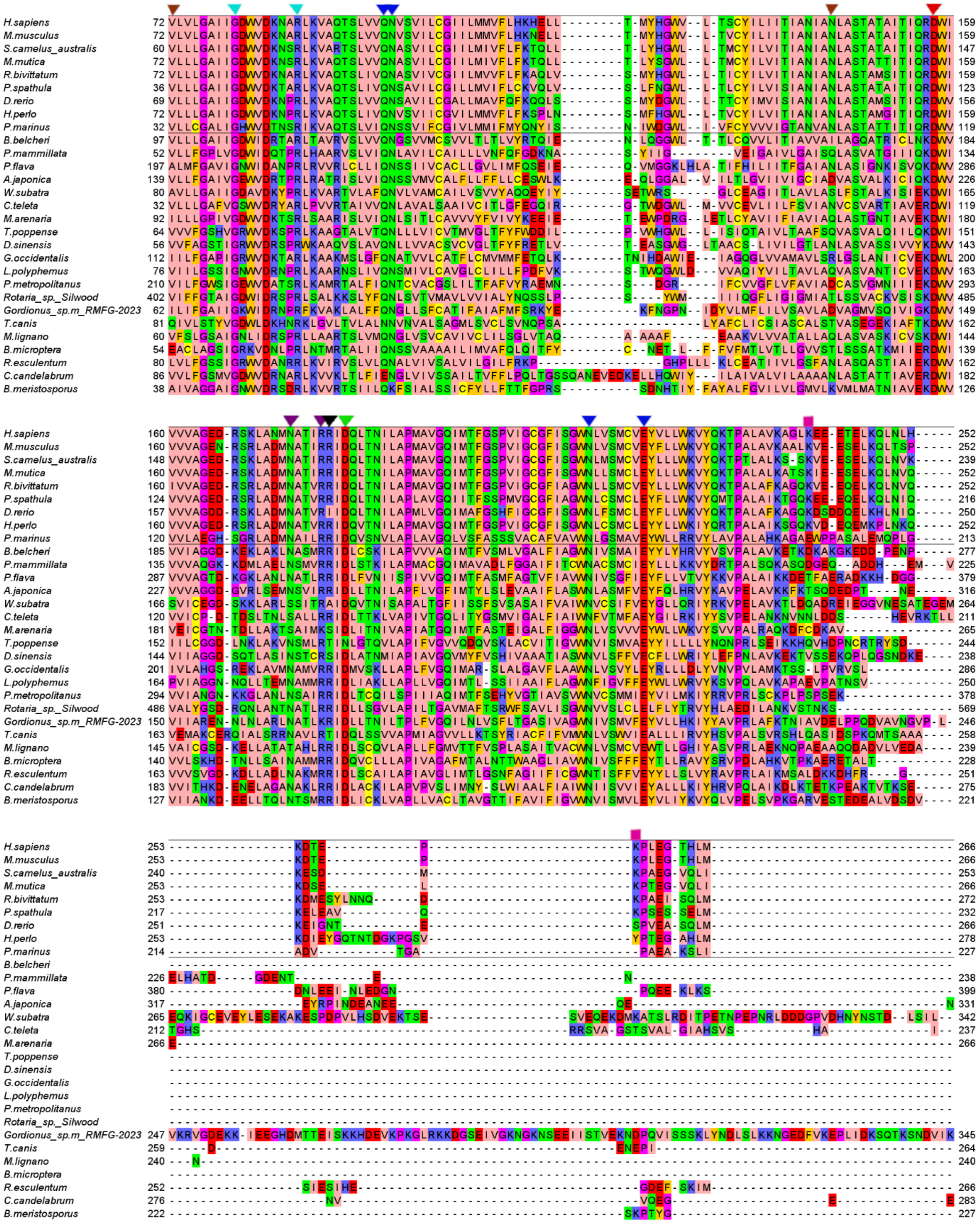
H.sapiens
M.musculus
S.camelus_australis
M.mutica
R.bivittatum
P.spathula
D.rerio
H.perlo
P.marinus
B.belcheri
P.mammillata
P.flava
A.japonica
W.subatra
C.teleta
M.arenaria
T.poppense
D.sinensis
G.occidentalis
L.polyphemus
P.metropolitanus
Rotaria_sp._Silwood
Gordionus_sp.m_RMFG-2023
T.canis
M.lignano
B.microptera
R.esculentum
C.candelabrum
B.meristosporus

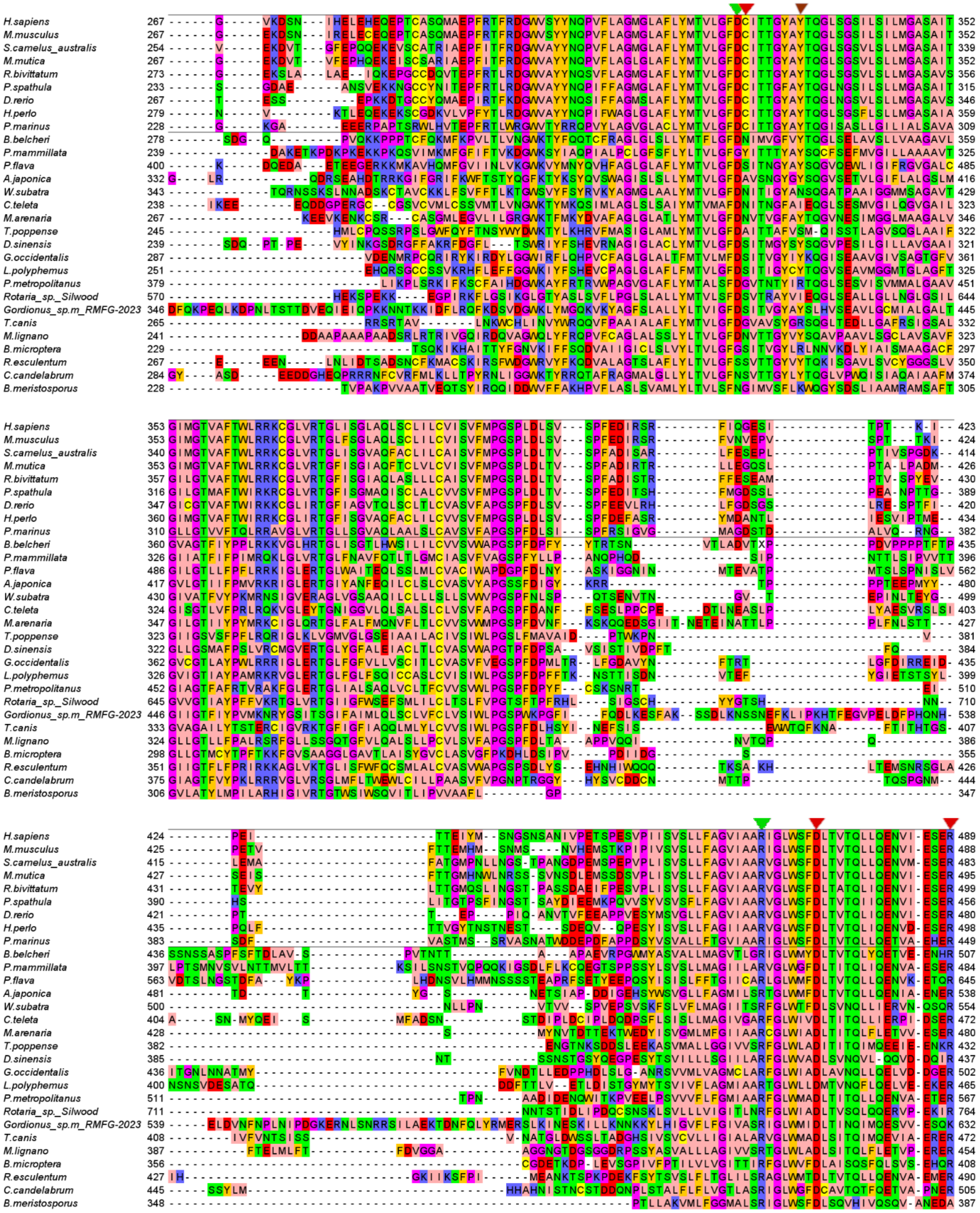
H.sapiens
M.musculus
S.camelus_australis
M.mutica
R.bivittatum
P.spathula
D.rerio
H.perlo
P.marinus
B.belcheri
P.mammillata
P.flava
A.japonica
W.subatra
C.teleta
M.arenaria
T.poppense
D.sinensis
G.occidentalis
L.polyphemus
P.metropolitanus
Rotaria_sp._Silwood
Gordionus_sp.m_RMFG-2023
T.canis
M.lignano
B.microptera
R.esculentum
C.candelabrum
B.meristosporus

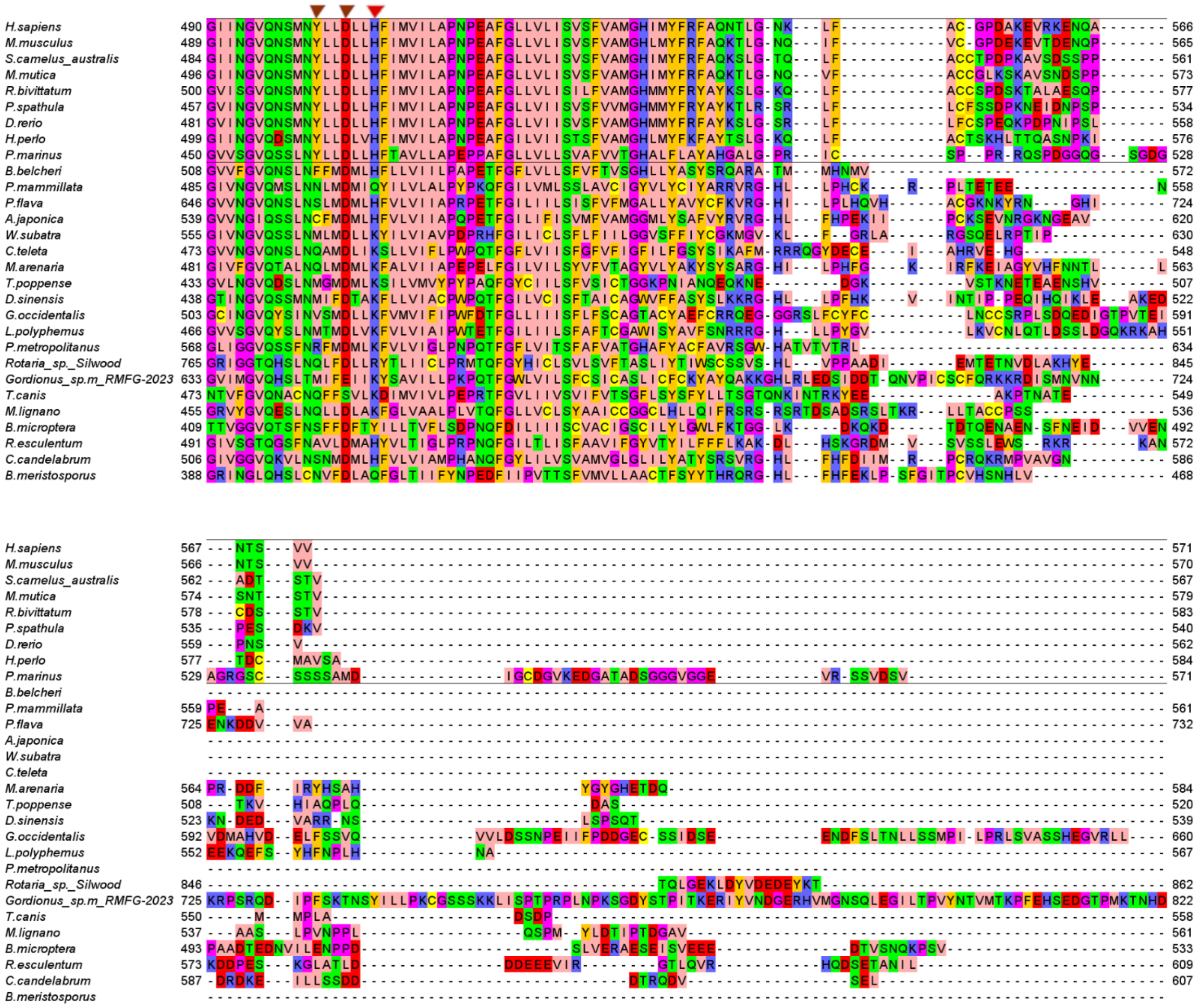

Figure S1. Multiple sequence alignment of SLC40A1 homologs in the Animalia and Fungi kingdoms. Organisms included in the range delimited by the two solid black lines are the most representatives of the Vertebrates subphylum.

Inward-open

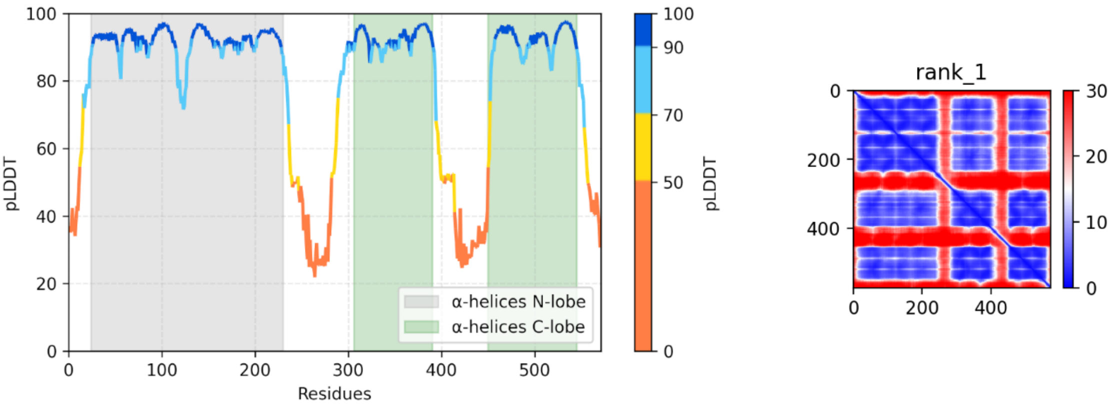

Occluded

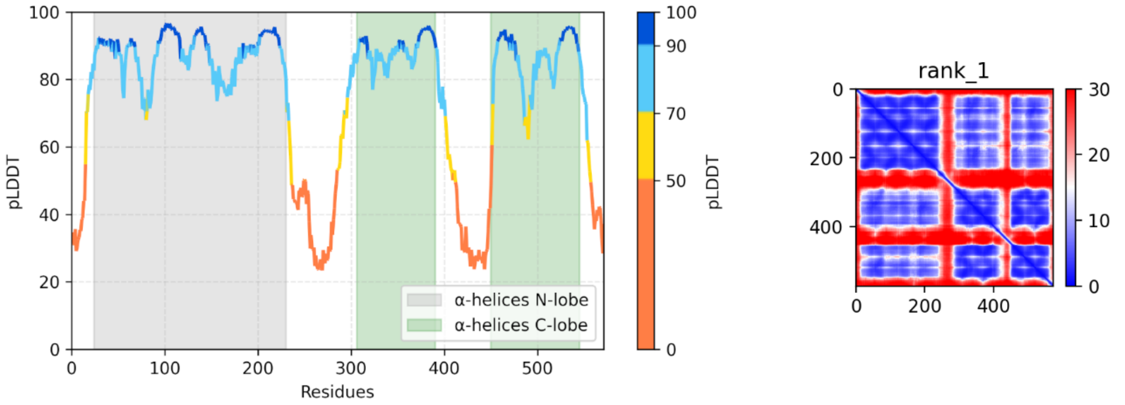

Outward-open

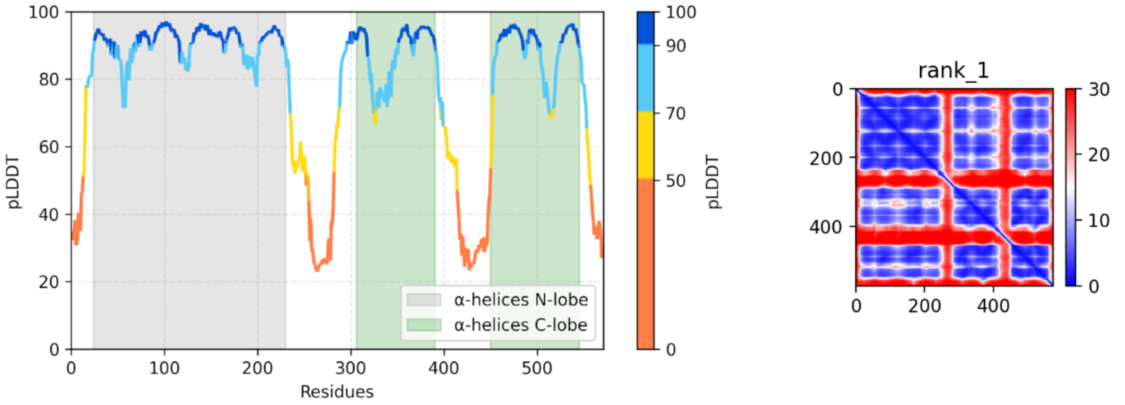

Figure S2. Representation of the ColabFold pLDDT and PAE metric values plotted for each hFPN conformational model.

Table S2. Quantity and proportions of lipids used to construct the plasma membrane in simulations of hFPN structural models.

CHARMM lipids	Outer leaflet	Inner leaflet	Area
POPC	24,0	10,5	57,0
PLPC	33,0	16,5	57,3
PAPE	4,5	18,0	58,0
POPE	4,5	21,0	55,8
POPI	0	7,5	57,0
PAPS	0	16,5	58,3
POPA	0	1,5	56,0
SSM	16,5	7,5	49,9
NSM	16,5	7,5	50,0
CMH	6,0	0	47,3
CHOL	55,5	43,5	28,0

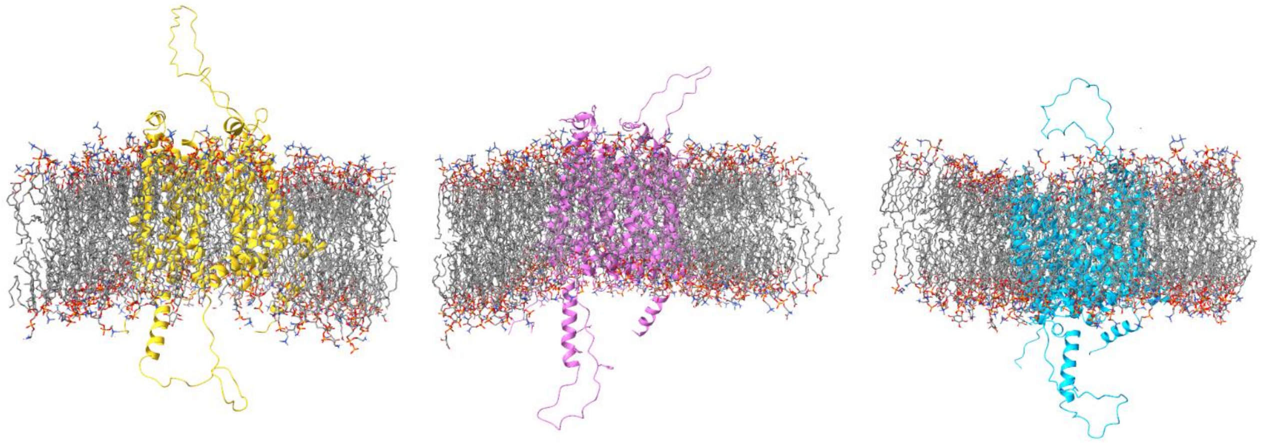

Figure S3. Representation of the initial structures of each hFPN conformation inserted in their own plasma membrane model: from the left, **inward–open (yellow), occluded (orchid) and outward–open conformations (deep sky blue).**

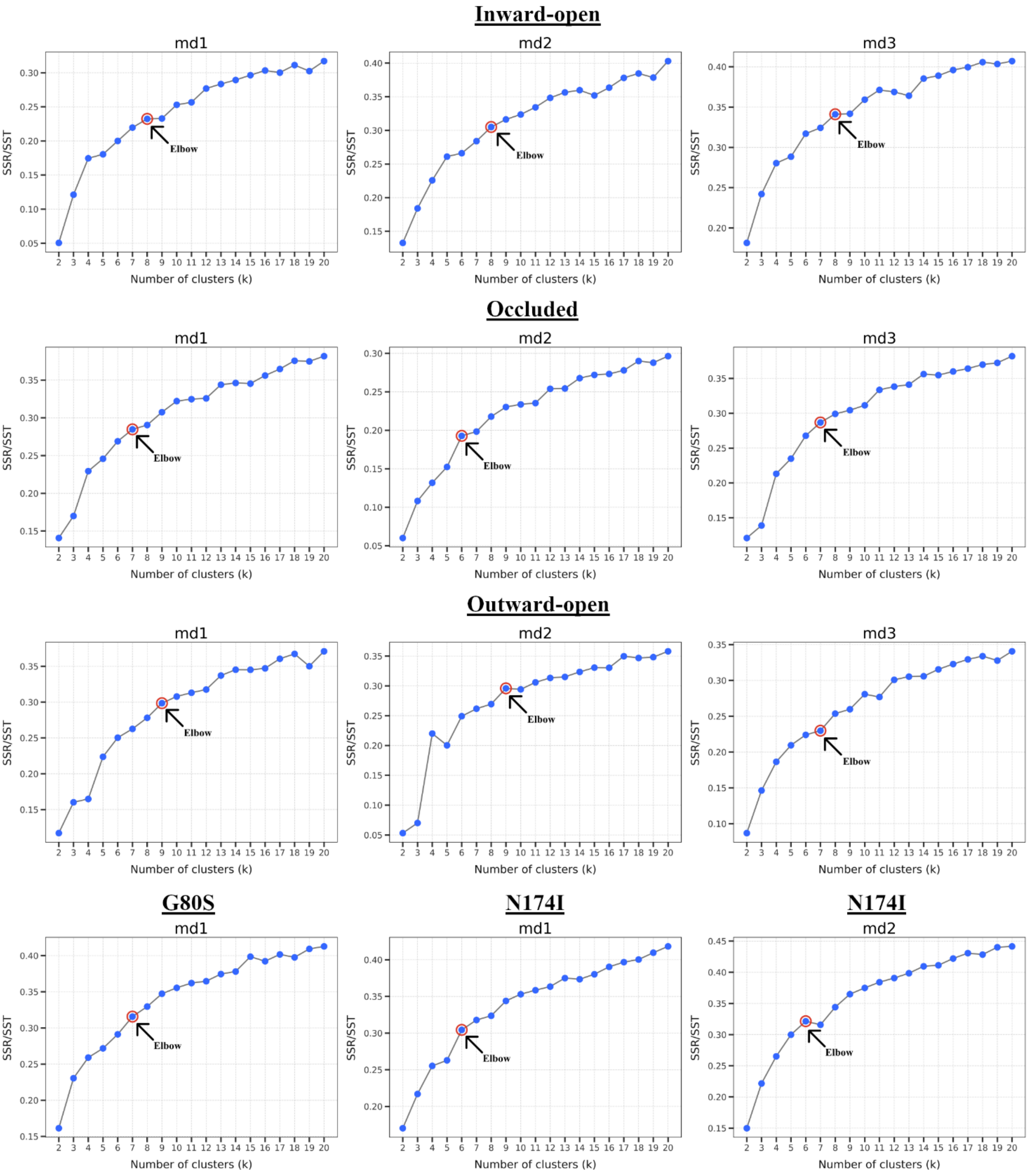

Figure S4: Representation of the SSR/SST value (Y axis) plotted against the number of clusters (X axis) for each MD simulation of each hFPN system.

Table S3. TM-align results from the comparison of our hFPN structural models with the experimentally determined structures of Ferroportin homologs and other MFS/SLC transporters.

Inward–open

Transporter (organism)	XylE (E. coli)	PepTSo (S. oneidensis MR-1)	**BbFPN (B. bacteriovorus)**	STP10 (A. thaliana)	SPNS2 (H. sapiens)	FLVCR1 (H. sapiens)
**PDB code**	4JA4	4UVM	5AYO	7AAR	8EX6	8QCT
**TM-score**	0,75809	0.77570	0,90866	0,73996	0,78483	0,74847

Occluded

Transporter (organism)	LacY G46W,G262 W (E. coli)	NarK (E. coli str. K-12 substr. MG1655)	PepTSt (S. thermophilus LMG 18311)	**Ferroportin (H. sapiens)**	OxlT (Oxalobacter formigenes)	VMAT2 (H. sapiens)
**PDB code**	4OAA	4U4W	5OXP	8BZY	8HPK	8THR
**TM-score**	0,74601	0,78617	0,77195	0,86959	0,75866	0,76208

Outward–open

Transporter (organism)	FucP (E. coli K-12)	GLUT5 (R. norvegicus)	**Ferroportin (H. sapiens)**	SPNS2 (H. sapiens)	OxlT (Oxalobacter formigenes)	FLVCR2 (H. sapiens)
**PDB code**	3O7Q	4YBQ	6W4S	8EX5	8HPJ	8QCY
**TM-score**	0,80095	0,782	0,96628	0,83674	0,77697	0,82796

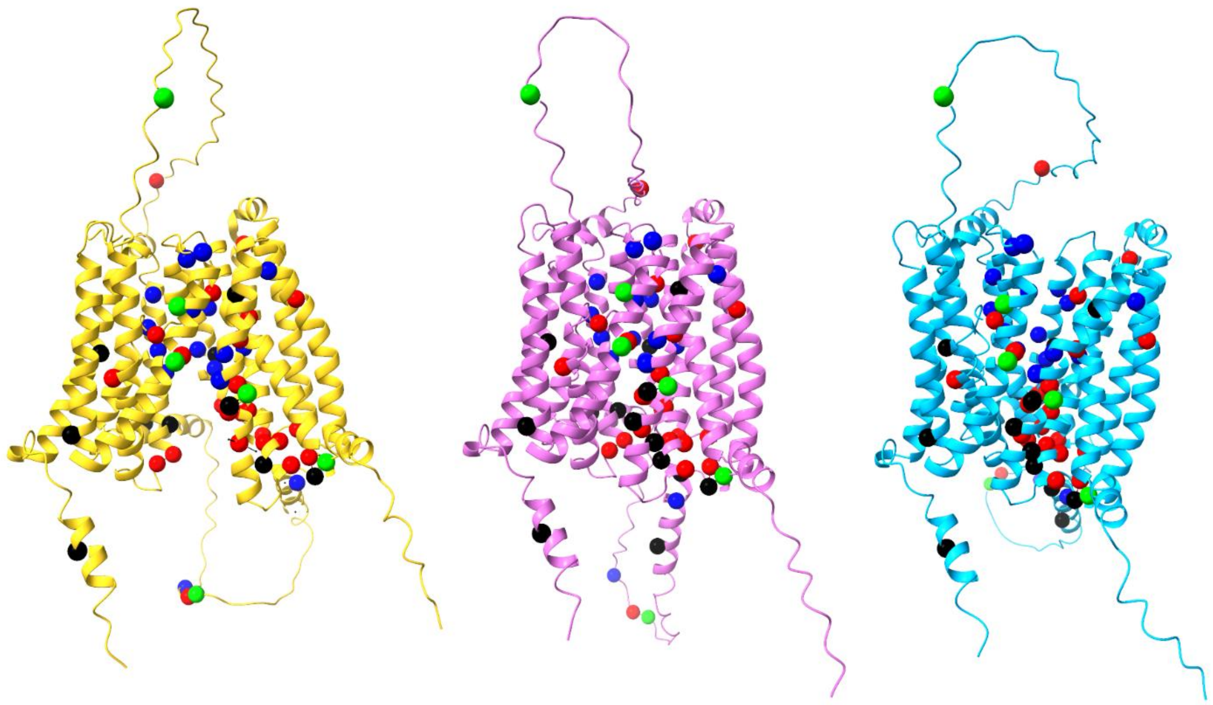

Figure S5. Representation of the three structural models of the inward-open (left), occluded (middle) and outward-open (right) conformations of hFPN. The spheres represent the pathogenic mutations known to cause Ferroportin–related iron overload, distinguished between "loss-of-function" (red spheres), "gain-of-function" (blue spheres), neutral (green spheres) and of uncertain significance (black spheres).

Inward-open

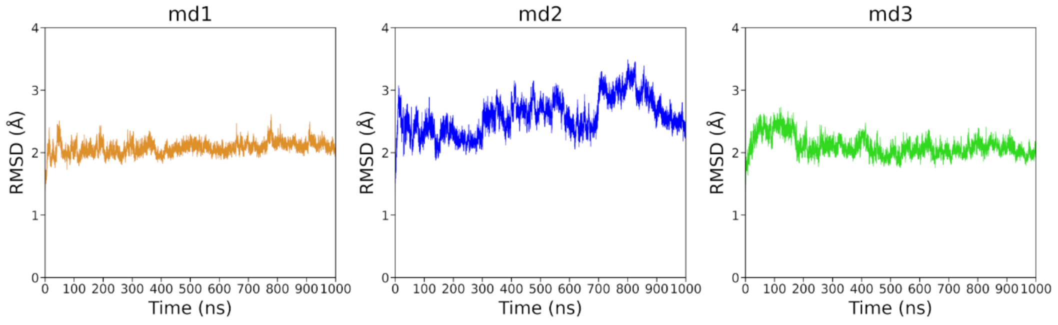

Occluded

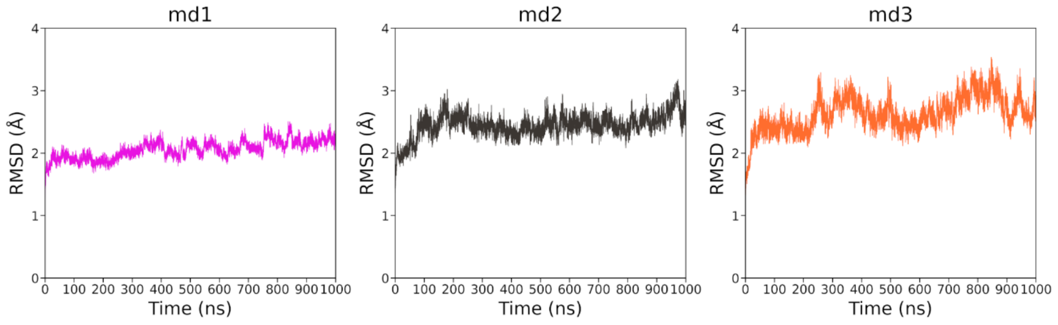

Outward-open

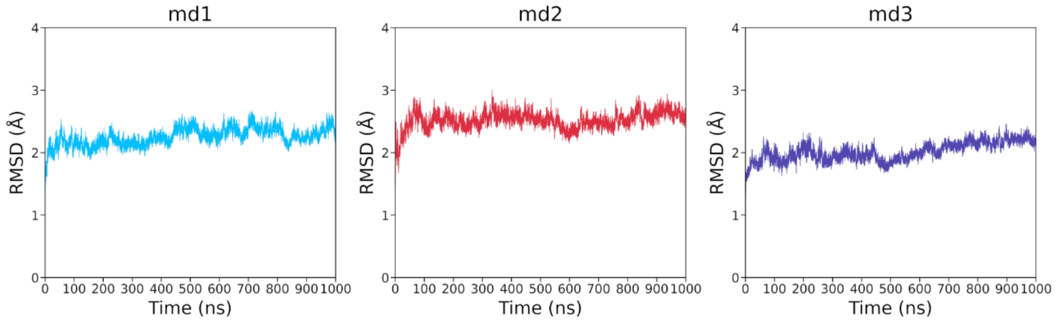

G80S N174I N174I

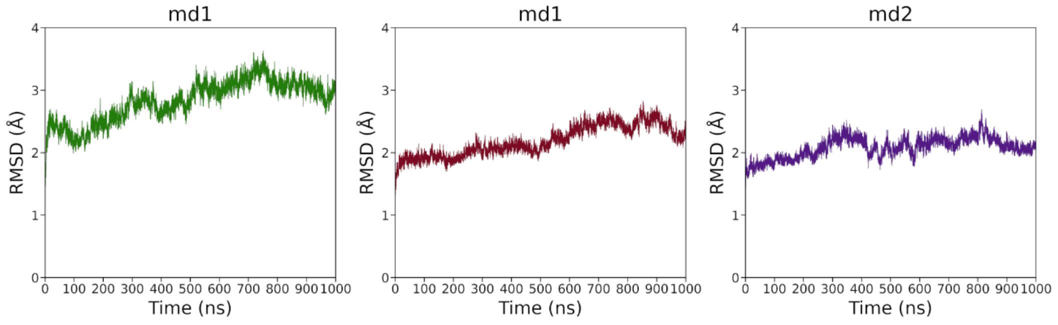

Figure S6: Backbone RMSD values calculated on the three replicas of the MD trajectories of each of the analysed systems.

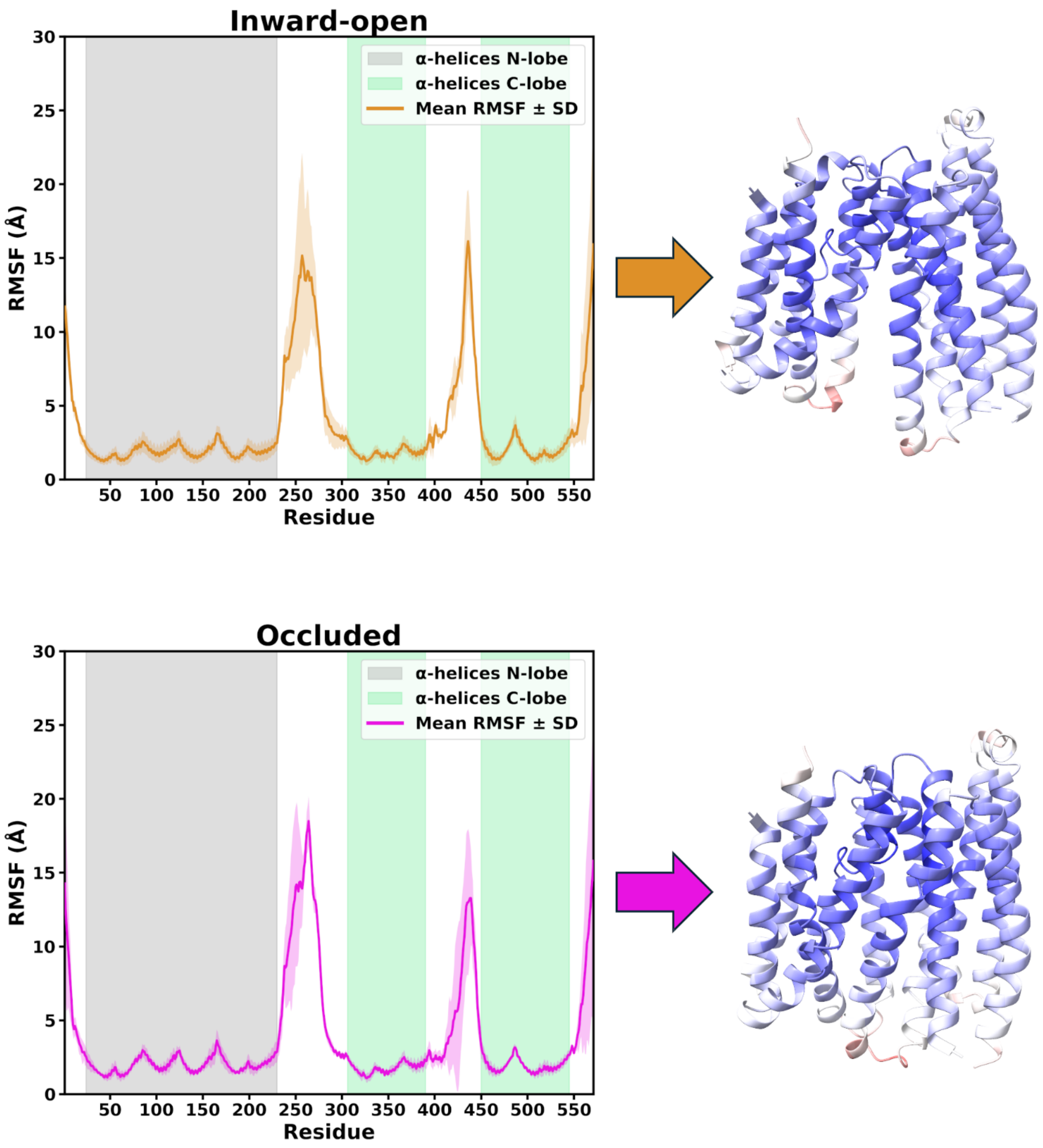

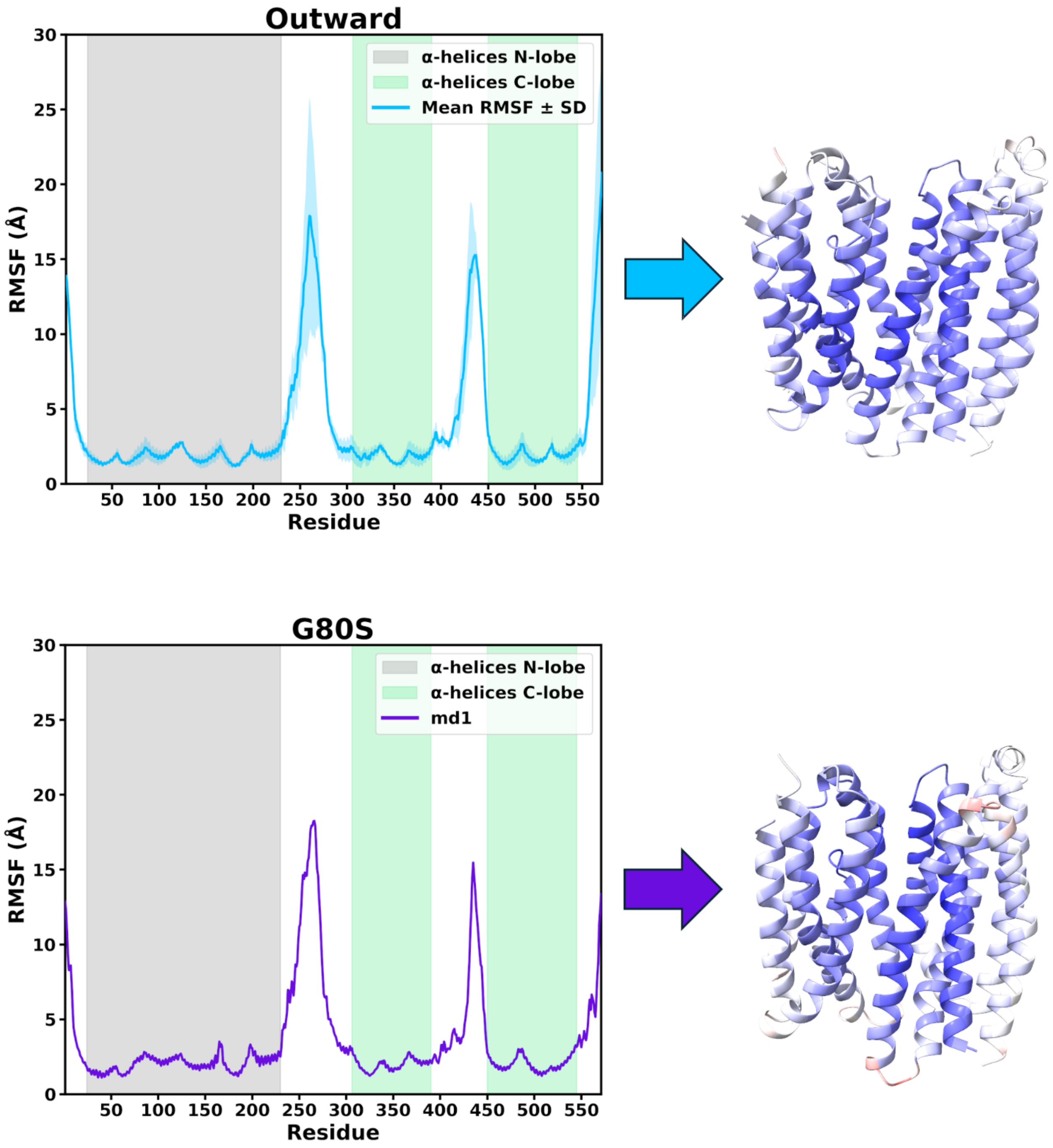

Outward
α-helices N-lobe
α-helices C-lobe
Mean RMSF ± SD
RMSF (Å)
Residue
0
5
10
15
20
25
30
50 100 150 200 250 300 350 400 450 500 550
G80S
α-helices N-lobe
α-helices C-lobe
md1
RMSF (Å)
Residue
0
5
10
15
20
25
30
50 100 150 200 250 300 350 400 450 500 550

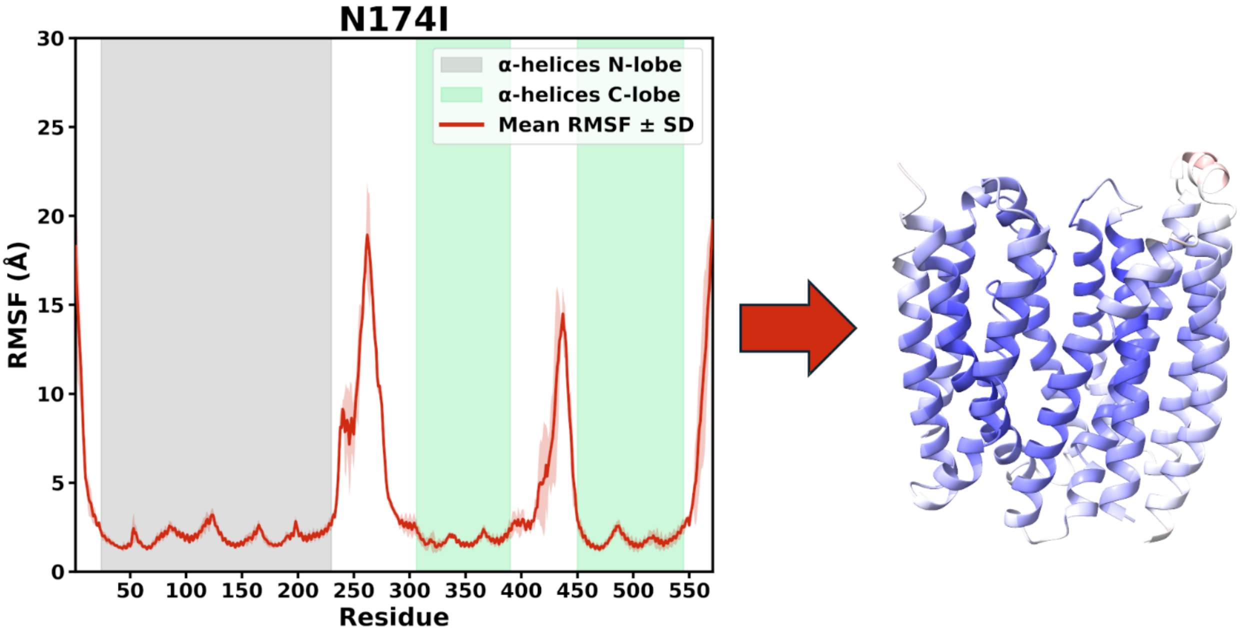

Figure S7: Representation of the mean RMSF values with standard deviation from the MD simulations of the analysed hFPN wild-type and mutant systems from a graphical (left) and from a structural point of view (right).
